# Identification of Merkel cells associated with neurons in engineered skin substitutes after grafting to full thickness wounds

**DOI:** 10.1371/journal.pone.0213325

**Published:** 2019-03-05

**Authors:** Jennifer M. Hahn, Kelly A. Combs, Christopher M. Lloyd, Kevin L. McFarland, Steven T. Boyce, Dorothy M. Supp

**Affiliations:** 1 Research Department, Shriners Hospitals for Children – Cincinnati, Cincinnati, Ohio, United States of America; 2 Department of Surgery, University of Cincinnati College of Medicine, Cincinnati, Ohio, United States of America; Massachusetts General Hospital, UNITED STATES

## Abstract

Engineered skin substitutes (ESS), prepared using primary human fibroblasts and keratinocytes with a biopolymer scaffold, were shown to provide stable closure of excised burns, but relatively little is known about innervation of ESS after grafting. This study investigated innervation of ESS and, specifically, whether Merkel cells are present in healed grafts. Merkel cells are specialized neuroendocrine cells required for fine touch sensation in skin. We discovered cells positive for keratin 20 (KRT20), a general marker for Merkel cells, in the basal epidermis of ESS after transplantation to mice, suggesting the presence of Merkel cells. Cells expressing KRT20 were not observed in ESS *in vitro*. However, widely separated KRT20-positive cells were observed in basal epidermis of ESS by 2 weeks after grafting. By 4 weeks, these cells increased in number and expressed keratins 18 and 19, additional Merkel cells markers. Putative Merkel cell numbers increased further between weeks 6 and 14; their densities varied widely and no specific pattern of organization was observed, similar to Merkel cell localization in human skin. KRT20-positive cells co-expressed epidermal markers E-cadherin and keratin 15, suggesting derivation from the epidermal lineage, and neuroendocrine markers synaptophysin and chromogranin A, consistent with their identification as Merkel cells. By 4 weeks after grafting, some Merkel cells in engineered skin were associated with immature afferents expressing neurofilament-medium. By 8 weeks, Merkel cells were complexed with more mature neurons expressing neurofilament-heavy. Positive staining for human leukocyte antigen demonstrated that the Merkel cells in ESS were derived from grafted human cells. The results identify, for the first time, Merkel cell-neurite complexes in engineered skin *in vivo*. This suggests that fine touch sensation may be restored in ESS after grafting, although this must be confirmed with future functional studies.

## Introduction

Burns are relatively common injuries, accounting for over 350,000 emergency room visits in the United States annually [1]. Advances in burn care have contributed to reduced mortality rates in recent decades, which has caused a shift in focus from acute burn care towards optimizing the long-term quality of wound healing for improved functional outcomes [2]. Although small and superficial partial-thickness wounds can heal without grafting, deep partial-thickness and full-thickness burns generally require skin grafting to achieve wound closure [3]. The prevailing standard for skin grafting is split-thickness skin autograft taken from uninjured donor skin of the same patient [2, 3]. Burn scars, including scarring of grafted burns and donor sites, can result in substantial morbidity in addition to cosmetic impairment. Pain, itch, abnormal pigmentation, and restricted range of motion due to contraction of burn scars are common problems that impair quality of life for burn survivors [2]. In particular, itch is considered a major problem that contributes significantly to reduced quality of life in burn survivors [4, 5]. Itch is present in most pediatric burn patients at the time of discharge and a majority still experience itching two years after injury [6]; similar trends have been reported in the adult burn population [7]. Neuronal damage and nervous system morbidity, including sensory loss, is also commonly reported in patients after burn injury [8–10]. Clinical studies have documented significant deficits in sensations including touch, heat, and cold in grafted burns [10–15]. In particular, grafted burns exhibit significant sensory deficits and reductions in innervation density compared with uninjured skin, which may persist for years after initial injury [11, 15, 16]. In one study of survivors of large burns affecting over 30% total body surface area (TBSA), one third of subjects reported sensory loss at 20–30 years after injury [15]. Sensory loss was also reported in patients with smaller burns, even in “good quality” scars [14].

For patients with very large full-thickness burns covering more than 50% total body surface area (TBSA), donor autograft skin may be limited, delaying wound closure and complicating recovery [2]. For these patients, tissue engineered skin replacements have been developed to meet the need for prompt wound closure. Cultured epithelial autografts (CEA) have been used for several decades as an adjunctive treatment in patients with very large burns [17, 18]. Comprised of cultured autologous keratinocytes, CEA have a relatively simple structure; cells are cultured *in vitro* until a multilayer epithelium is formed, and this epithelial sheet is transplanted to an excised burn wound [19]. The use of CEA has been shown to facilitate wound closure and can potentially improve survival rates [20–22]. However, because CEA only replaces the epidermis, it exhibits deficiencies compared with split-thickness autograft, which also has a dermal component and basement membrane. Deficiencies include fragility, blistering, and sensitivity to mechanical shear after transplantation, all of which contribute to reduced engraftment rates [20]. These deficiencies can be overcome by incorporating a dermal component into the engineered tissue *in vitro*. A bilayer skin replacement, referred to as “Engineered Skin Substitutes” (ESS), is comprised of a collagen-based scaffold populated with autologous fibroblasts and keratinocytes, enabling replacement of both epithelial and connective tissues in a single surgical procedure [23]. During *in vitro* incubation, keratinocytes in ESS form a multilayered, stratified epidermal substitute, while fibroblasts proliferate and begin to remodel the dermal component. Importantly, interactions between fibroblasts and keratinocytes enable deposition of basement membrane components in ESS *in vitro*, prior to grafting, which minimizes the mechanical fragility and blister formation observed with CEA [23]. In clinical studies, ESS were shown to provide stable, long-term closure of excised full-thickness burns [24, 25]. In pediatric burn patients with >50% total body surface area burns, ESS significantly decreased the requirement for harvesting of donor autograft and reduced mortality [25]. In this pediatric patient population, ESS were shown to grow with the patient with limited contraction, and displayed relatively little scarring [24, 25]. However, relatively little is known about sensory function or innervation of ESS after grafting.

In uninjured human skin, specialized epidermal cells called Merkel cells are involved in fine touch sensation [26, 27]. Merkel cells are present in the basal layer of the epidermis where they interact with nerves to form Merkel cell-neurite complexes [26–28]. Studies in mice have demonstrated that Merkel cells actively participate in mechanotransduction and are required for fine touch sensation [27, 29]. Recent studies showed that deletion of Piezo2, a mechanically activated channel present in Merkel cells, not only impaired touch sensation in mice but also sensitized them to mechanical pain [30]. In other studies in mice, increased itch in aging and dry skin was found to be correlated with loss of Merkel cells, and ablation of Piezo2 function in Merkel cells increased itch behavior [31]. Thus, Merkel cells may regulate the balance between fine touch sensation and pain and itch. Although these cells are difficult to distinguish from epidermal keratinocytes in routine histological sections, Merkel cells can be identified by immunohistochemical markers, as they express specific keratins not normally found in keratinocytes. Keratin 20 (KRT20) is considered a specific marker for Merkel cells in skin because it is not expressed in epidermal keratinocytes; keratins 8 (KRT8), 18 (KRT18), and 19 (KRT19) are expressed in Merkel cells, but are also found in some keratinocytes of the outer root sheath of hair follicles and are thus considered less specific markers [32]. Merkel cells in mice are found in organized clusters in touch-sensitive regions, such as tips of digits and whiskers, and in structures called “touch domes” associated with hair follicles [28]. In humans, distribution of Merkel cells in skin of different body regions is highly variable, but they are found at highest densities in regions involved in tactile perception, such as the palms and fingertips [33]. Analysis of the spatial organization of human Merkel cells showed that they are not regularly distributed in human skin, although three basic patterns were observed: Merkel cells were arranged either in clumps, linear or “arciform” arrangements, or were found as scattered individual cells [33]. These patterns were not correlated with specific body locations, and all three patterns could be found in a single body site.

Although innervation of grafted skin and burn scars has been investigated, relatively little is known about Merkel cells in skin grafts. One study reported the observation of Merkel cells associated with intraepithelial nerve endings in postburn scar, but they were present in “very limited” amounts [34]. Another study examined localization of Merkel cells in meshed, split-thickness autograft and CEA grafted to excised burns or excised giant congenital nevi [35]. In that study, Merkel cells were observed in biopsies of healed split-thickness autograft from 2 of 2 patients in which the sole of the foot was used as the autograft donor site, but in only 3 of 22 non-sole autografts [35]. Degeneration of innervating neurites was observed by 4 weeks after grafting, and although Merkel cells were observed in biopsies collected up to 6 years after grafting, re-innervation was never observed [35]. Merkel cells were observed in healed CEA in 6 of 19 patients treated with sole keratinocyte-derived CEA, but not in CEA prepared with keratinocytes from other body sites [35]. None of the observed Merkel cells in CEA were associated with neurons [35].

To begin to address whether restoration of fine touch sensation can occur after transplantation of engineered skin containing both keratinocytes and fibroblasts, the current study investigated whether innervated Merkel cells are found in ESS after transplantation to full-thickness wounds.

## Materials and methods

### Primary cell culture

De-identified human trunk skin was obtained from a healthy 15-year-old donor undergoing elective plastic surgery at the Shriners Hospitals for Children–Cincinnati. This tissue sample was classified as “medical waste” by the attending surgeons. The University of Cincinnati Institutional Review Board determined that collection of de-identified medical waste does not meet the definition of human subjects research. This study (Study ID: 2013–4582) is therefore exempt from requirements for informed consent according to 45CFR46.101(b)(4) [36].

Primary fibroblasts and keratinocytes were isolated and cultured as previously described [37–39]. Full-thickness skin was cut to narrow strips (2–3 mm wide), which were incubated overnight at 4° C in Dispase (Roche Life Sciences, Indianapolis, IN) to separate dermal and epidermal layers. Dermal pieces were finely minced, incubated at 37° C in Collagenase (Worthington Biochemical Corp., Lakewood, NJ) for one hour, rinsed in culture media, and transferred to tissue culture flasks. Fibroblasts were cultured in Gibco DMEM (Low Glucose; Thermo Fisher Scientific) supplemented with 10 ng/mL epidermal growth factor (EGF; Pepro-Tech, Rocky Hill, NJ), 5 μg/ml human insulin (Sigma-Aldrich; St. Louis, MO, USA), 0.5 mg/mL hydrocortisone (Sigma-Aldrich), 0.1 mM ascorbic acid-2-phosphate (AA2P; Sigma-Aldrich), 4% fetal bovine serum (FBS; Gibco/Thermo Fisher Scientific, Waltham, MA), and 1X penicillin-streptomycin-fungizone (PSF; Thermo Fisher Scientific). Epidermal pieces were incubated for five minutes at 37° C in 0.025% trypsin, 0.02% EDTA (Sigma-Aldrich, St. Louis, MO) to release keratinocytes. The cell suspension was filtered through a 70 μm cell strainer (BD Falcon, Bedford, MA) and trypsin was neutralized with 10% FBS. Keratinocytes were centrifuged, resuspended in keratinocyte growth medium and inoculated into flasks coated with recombinant human type I collagen (Coating Matrix; Invitrogen/Thermo Fisher Scientific). Keratinocyte medium consisted of modified MCDB153 prepared in-house [39] with 0.06 mM calcium chloride and supplemented with 0.2% bovine pituitary extract (Hammond Cell Tech, Windsor, CA), 1 ng/ml EGF, 5 μg/ml human insulin, 0.5 mg/ml hydrocortisone, and 1X PSF. All cells were harvested when they reached 80–90% confluence and were cryopreserved in their respective growth medium containing 10% dimethyl sulfoxide and 20% FBS using a controlled rate freezer. Cells were recovered from storage in liquid nitrogen using the same culture medium as for initial culture except that the calcium chloride concentration of the keratinocyte medium was increased to 0.2 mM, and the collagen coating of the flasks was discontinued. Cells were expanded through one additional passage prior to preparation of engineered skin.

### Preparation of engineered skin substitutes (ESS)

ESS were prepared essentially as previously described [39] with the following modifications. Fibroblasts were inoculated at 0.5 X 10^6^/cm^2^ onto sterile collagen-glycosaminoglycan scaffolds supported at the air-liquid interface using polyvinyl acetal sponges. Keratinocytes were inoculated onto the dermal substrates 24 hours later at 1.0 X 10^6^/cm^2^ and ESS were transferred to cotton pads supported by perforated stainless steel platforms for incubation at the air-liquid interface (37°C, 5% CO_2_). Culture medium for ESS consisted of DMEM/F12 (Sigma-Aldrich) supplemented with 1 mM strontium chloride, 0.3% FBS, 1X ITS Supplement (Sigma Aldrich), 10 μg/ml linoleic acid, 0.1 mM AA2P, 20 pM triiodothyronine, 0.5 μg/ml hydrocortisone, 5 ng/ml keratinocyte growth factor (Peprotech, Rocky Hill NJ), 1 ng/ml basic fibroblast growth factor (Peprotech), and 1X PSF [37]. ESS were cultured *in vitro* for 10 days prior to transplantation to mice.

### Grafting to mice and collection of tissue samples

Animal studies were approved by the University of Cincinnati Institutional Animal Care and Use Committee. Immunodeficient mice (NIH-III-nude strain; Charles River Laboratories, Wilmington, MA) were used (n = 24) to enable engraftment of ESS containing human cells. In addition to carrying the *nude* mutation in the *Foxn1* gene, these mice also have mutations in *x-linked immune defect* (*xid*) and *beige* (*bg*) genes, resulting in defects of T cells, B cells, and natural killer (NK) cells, respectively. A full-thickness wound (2 cm X 2 cm) was prepared on the flank of each mouse, leaving the panniculus carnosus layer intact. ESS were cut to 2 cm X 2 cm squares and were sutured to the wounds with an overlying piece of N-terface (Winfield Laboratories, Richardson, TX, USA) to prevent adherence of dressing materials. Grafts were dressed using gauze coated with antimicrobial ointment (equal parts Nystatin, Bactroban, and Neosporin) and tie-over stents. Dressings were covered with Tegaderm Transparent Film Dressing (3M, St. Paul, MN) and the mice were wrapped with Coban self-adherent bandages (3M). Two mice died during the study period and were excluded from the analysis. Two mice per time point were euthanized at 2, 4, 6, 8, 10, and 12 weeks after grafting, and remaining mice were euthanized at 14 weeks. ESS were excised and biopsies were fixed in 10% formalin or were embedded frozen for cryosectioning using OCT Compound (Fisher HealthCare, Pittsburgh, PA). Remaining samples of excised ESS were incubated in Dispase overnight at 4° C to enable separation of epidermis from dermis. Epidermal sheets were stored at -20°C in methanol until evaluation by immunohistochemistry. Formalin-fixed sections were processed, paraffin-embedded, sectioned, and stained with Hematoxylin and Eosin (H&E) by the Shriners Hospitals for Children–Cincinnati Histology Special Shared Facility.

### Immunohistochemistry

Localization of specific antigens in cryosections and epidermal pieces was performed by immunohistochemistry (IHC) using routine procedures. Cryosections for IHC were prepared by the Shriners Hospitals for Children–Cincinnati Histology Special Shared Facility. Details about the primary and secondary antibodies used for IHC, including dilutions used, are presented in Table 1. The KRT20 antibody (Abcam, Cambridge, MA) was incubated with sections for 1 hour at room temperature. All other primary antibody incubations were performed overnight (16 hours) at 4° C. All fluorescent secondary antibodies were incubated with sections for 1 hour at room temperature. For the KRT19 mouse monoclonal antibody (ThermoFisher), the Mouse on Mouse (M.O.M.) Basic Kit (catalog # BMK-2202; Vector Laboratories, Burlingame, CA) was used to block background staining due to use of a secondary anti-mouse antibody in the *in vivo* ESS samples, which were excised from mice. The Alexa Fluor 594 Tyramide SuperBoost Kit, goat anti-rabbit IgG (catalog # B40925; ThermoFisher) was used for detection of primary antibody against synaptophysin. Vectashield Antifade Mounting Medium with DAPI (4′,6-diamidino-2-phenylindole; catalog #H-1200, Vector Laboratories) was used to mount coverslips and counterstain nuclei. Sections were viewed and photographed with an Eclipse 90i microscope equipped with a DS-Ri1 Digital Microscope Camera (Nikon Instruments Inc., Melville, NY). Z-stacking was used to improve depth of field of digital images; all images for a given antibody were collected using identical settings for each tissue section.

**Table 1:** Antibodies used for immunohistochemistry.

Antigen	Conjugate	Species	Company	Catalog #	Lot/Batch #	Antibody Registry #	Clonality	Dilution
*Primary Antibodies*								
Keratin 20	None	Guinea Pig	Abcam	ab192682	GR314080-5	NA	Polyclonal	1:1000
Keratin 18	None	Rabbit	Abcam	ab24561	GR92020-1	AB_1310117	Polyclonal	1:250
Keratin 19*	None	Mouse	ThermoFisher	MA1-91210	QD2006615	AB_1955705	Monoclonal	1:400
Human Leukocyte Antigen (HLA)-ABC	FITC	Mouse	Accurate Chemical	CLHLA01F	0131B	AB_10060657	Monoclonal	1:50
Keratin 15	None	Chicken	Bio Legend	PCK-153P	D14BF00272	AB_291540	Polyclonal	1:250
Collagen IV	None	Rabbit	Yo Proteins	507	60607	AB_2082817	Polyclonal	1:50
E-Cadherin	None	Rabbit	Novus	NB110-56937	NA	AB_843761	Monoclonal	1:200
Synaptophysin**	None	Rabbit	Abcam	ab32127	GR312544-10	AB_2286949	Monoclonal	1:400
Neurofilament Heavy	None	Chicken	Neuromics	CH22104	402107	AB_1619710	UNK	1:7500
Neurofilament Medium	None	Chicken	Abcam	ab39371	GR259030-4	AB_726980	Polyclonal	1:1000
*Secondary Antibodies*:								
Chicken IgG (H+L)	AF-594	Goat	Molecular Probes	A11042	1504513	AB_142803	UNK	1:400
Guinea Pig IgG (H+L)	AF-594	Goat	Novus	NBP1-75746	1841755	AB_11005097	UNK	1:400
Guinea Pig IgG (H+L)	AF-488	Goat	Molecular Probes	A11073	26-54-062312	AB_142018	UNK	1:400
Rabbit IgG (H+L)	AF-594	Donkey	Molecular Probes	A21207	1938375	AB_141637	UNK	1:400
Mouse IgG (H+L)	AF-594	Donkey	Molecular Probes	A21203	987237	AB_141633	UNK	1:400

Temp, temperature; FITC, fluorescein isothiocyanate; AF, Alexa Flour; NA, not available; UNK, unknown; RT, room temperature.

*For this antibody, the Mouse on Mouse Detection Kit (Vector Laboratories) was used for detection; see text for details.

**For this antibody, the Tyramide SuperBoost Kit (ThermoFisher) was used for signal amplification; see text for details.

### Flow cytometry and statistical analysis

Keratinocytes of the same strain used for preparation of ESS, and a second unrelated strain from an unrelated 13-year-old donor were recovered from cryopreservation and inoculated into tissue culture flasks at 3,000 cells/cm^2^. When cells reached ~90% confluence, they were harvested by trypsinization (passage 1); a portion were fixed and stored for analysis by fluorescence-assisted cell sorting (FACS), and the remainder were inoculated into fresh flasks at 3,000 cells/cm^2^. When these cells reached ~90% confluence, they were harvested (passage 2) for FACS analysis. Cells were stained with propidium iodide (Thermo Fisher), 1μL (50 ng) per 1 x 10^6^ cells, to enable determination of cell viability. Cells were subsequently fixed in 2% paraformaldehyde and stored in 50% methanol at -20° C. To stain KRT20-positive keratinocytes, cells were permeabilized with 0.1% Triton X-100, incubated with 2% bovine serum albumin to block non-specific binding, and labeled with Alexa Fluor 647-conjugated anti-KRT20 mouse monoclonal antibody (NBP2-42616AF647; NOVUS), 1μl (0.7 μg) per 1 x 10^6^ cells. FACS analysis was performed at the Shriners Hospitals for Children–Cincinnati Flow Cytometry Special Shared Facility using a Becton Dickinson LSRII analyzer and 640 nm laser. Statistical analysis was used to compare the proportion of cells positive for KRT20 at passage one and passage two using a paired t-test (SAS Statistical Analysis Software version 9.4; SAS Institute, Inc., Cary, NC). Statistical significance was considered at p< 0.05.

## Results

### Identification of Merkel cells in engineered skin after transplantation to mice

After 10 days of incubation *in vitro*, keratinocytes in ESS formed a stratified epidermis with a well-organized basal cell layer and cornified surface (S1 Fig). As early as two weeks after transplantation, grafts appeared well adhered to the wound margins and displayed a dry surface (S2 Fig). At this time point, fibroblasts had begun to remodel the dermal scaffold and produce newly synthesized collagen. By 10–14 weeks after grafting, the morphologic reticulations of the bovine collagen scaffold were no longer observed, having been replaced by newly synthesized connective tissue (S1 Fig). Epidermal organization increased after transplantation, with rete ridge-like structures visible by 4–6 weeks after grafting (S1 Fig).

**Fig 1:**
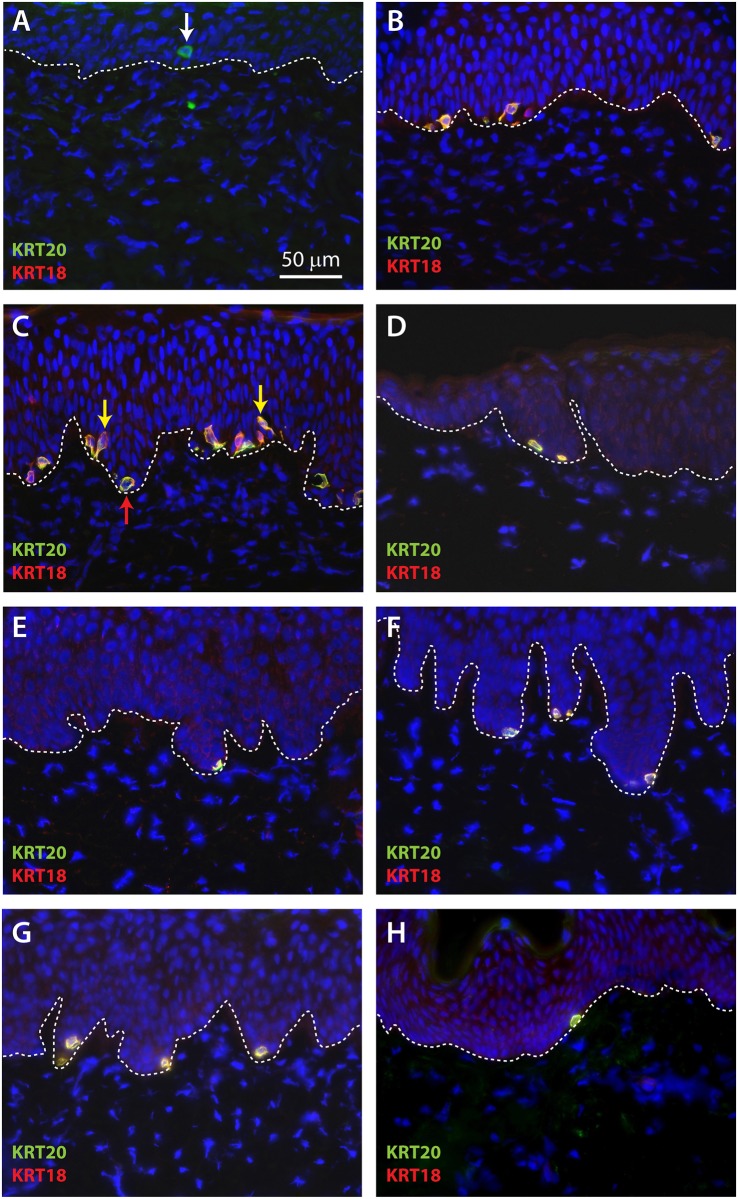
Localization of Merkel cells in cross-sections of ESS after grafting to mice. Immunohistochemistry was performed using antibodies against KRT20 (green) and KRT18 (red); DAPI was used to counterstain nuclei (blue). Shown are representative sections of ESS excised from mice at week 2 (**A**), week 4 (**B**), week 6 (**C**), week 8 (**D**), week 10 (**E**), week 12 (**F**), and week 14 (**G**) after grafting. **H**, Section of normal human skin (control). Dashed lines indicate locations of dermal-epidermal junctions. Rare KRT20-positive cells were observed at 2 weeks after grafting (arrow in A); co-localization of KRT20 and KRT18 was observed at later time points (**B-G**). Examples of oval (red arrow) and dendritic (yellow arrows) Merkel cell shapes are indicated (**C**). Scale bar in **A** (50 μm) is same for all panels.

Immunohistochemistry was used to identify putative Merkel cells in sections of ESS. KRT20 is the most widely accepted marker used for identification of Merkel cells; KRT18 has also been used to identify Merkel cells [32, 40–42]. No cells staining positive for KRT20 were identified in ESS *in vitro*. At two weeks after grafting, rare cells positive for KRT20 were observed in sections of ESS at or near the dermal-epidermal junction (Fig 1A). By four weeks after grafting, multiple cells positive for both KRT20 and KRT18 were observed in the basal epidermis of ESS (Fig 1B–1G). Although somewhat more numerous at week 4 compared with week 2, these cells were often difficult to find and many sections were observed that did not contain any KRT20/KRT18-positive cells. The numbers of KRT20/KRT18-positive cells appeared to increase at later time points after grafting, and most of these cells were localized at the tips of the rete ridge-like structures in ESS (Fig 1C–1G). Merkel cells of different shapes, including round or oval cells as well as dendritic cells, were noted in ESS *in vivo* (Fig 1). This is consistent with previous observations of Merkel cells in humans [26] and rodents [43–45]. KRT20 and KRT18 exhibited a perinuclear localization pattern in Merkel cells, although they were also localized to cellular projections in dendritic Merkel cells. The perinuclear localization pattern has been observed previously in normal Merkel cells and is also a feature of KRT20-positive cells in Merkel cell carcinoma [46].

To confirm that the appearance of KRT20/KRT18-positive cells in ESS after grafting was not specific for the donor cell strain used for the current study, we analyzed archived samples of ESS from previous experiments. ESS prepared with cells isolated from a 33 year old donor and, separately, a 15-year-old donor, were examined after grafting to immunodeficient mice. Analysis of biopsies of ESS collected at 15 weeks after grafting revealed the presence of KRT20/KRT18-positive cells at densities similar to those observed in the current study (S3 Fig). Because cells in the basal epidermis expressing KRT20 and KRT18 are considered to be Merkel cells, we will hereafter refer to the KRT20/KRT18-positive cells identified in ESS after grafting as Merkel cells.

**Fig 2:**
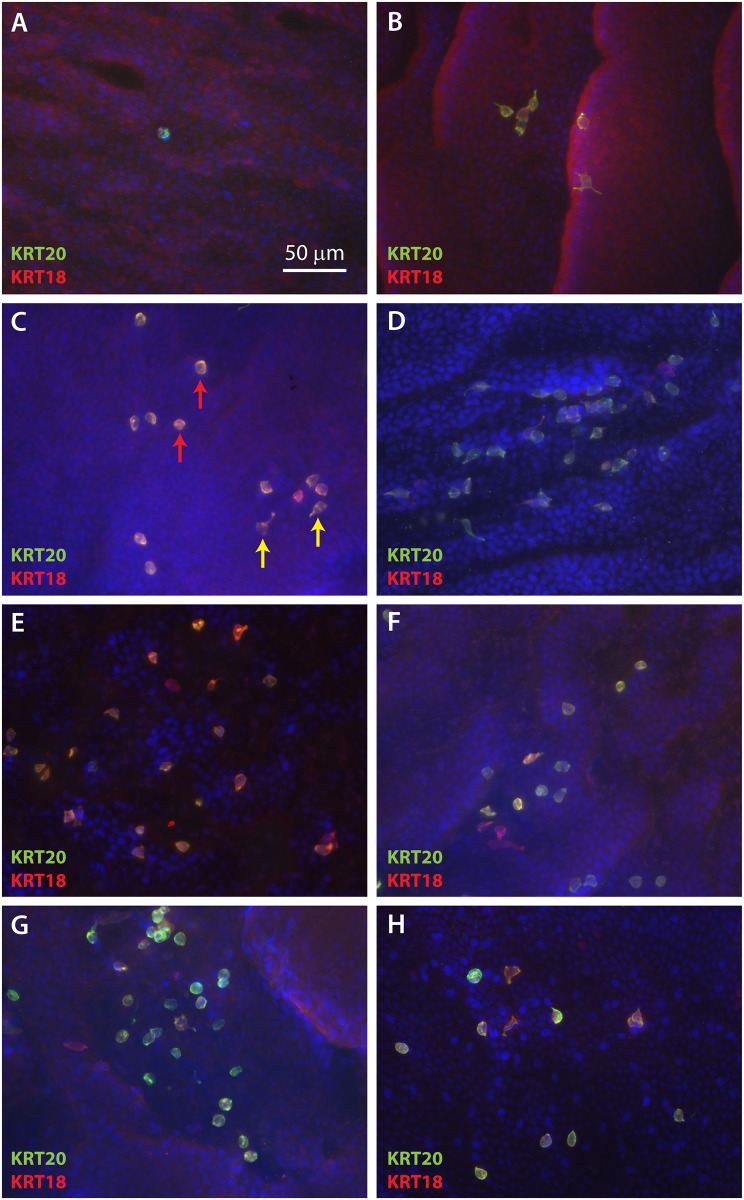
Localization of Merkel cells in ESS after grafting suggests random organization. Epidermal sheets of ESS excised from mice at 2 weeks (**A**), 4 weeks (**B**), 6 weeks (**C**), 8 weeks (**D**), 10 weeks (**E**), 12 weeks (**F**), and 14 weeks (**G**) after grafting, and normal human skin (**H**) are shown following immunostaining with antibodies against KRT20 (green) and KRT18 (red). DAPI was used to counterstain nuclei (blue). Representative *en face* images are shown such that the basal epidermis is viewed from the dermal side. Examples of oval (red arrow) and dendritic (yellow arrows) Merkel cell shapes are indicated (**C**). Scale bar in **A** (50 μm) is same for all panels.

**Fig 3:**
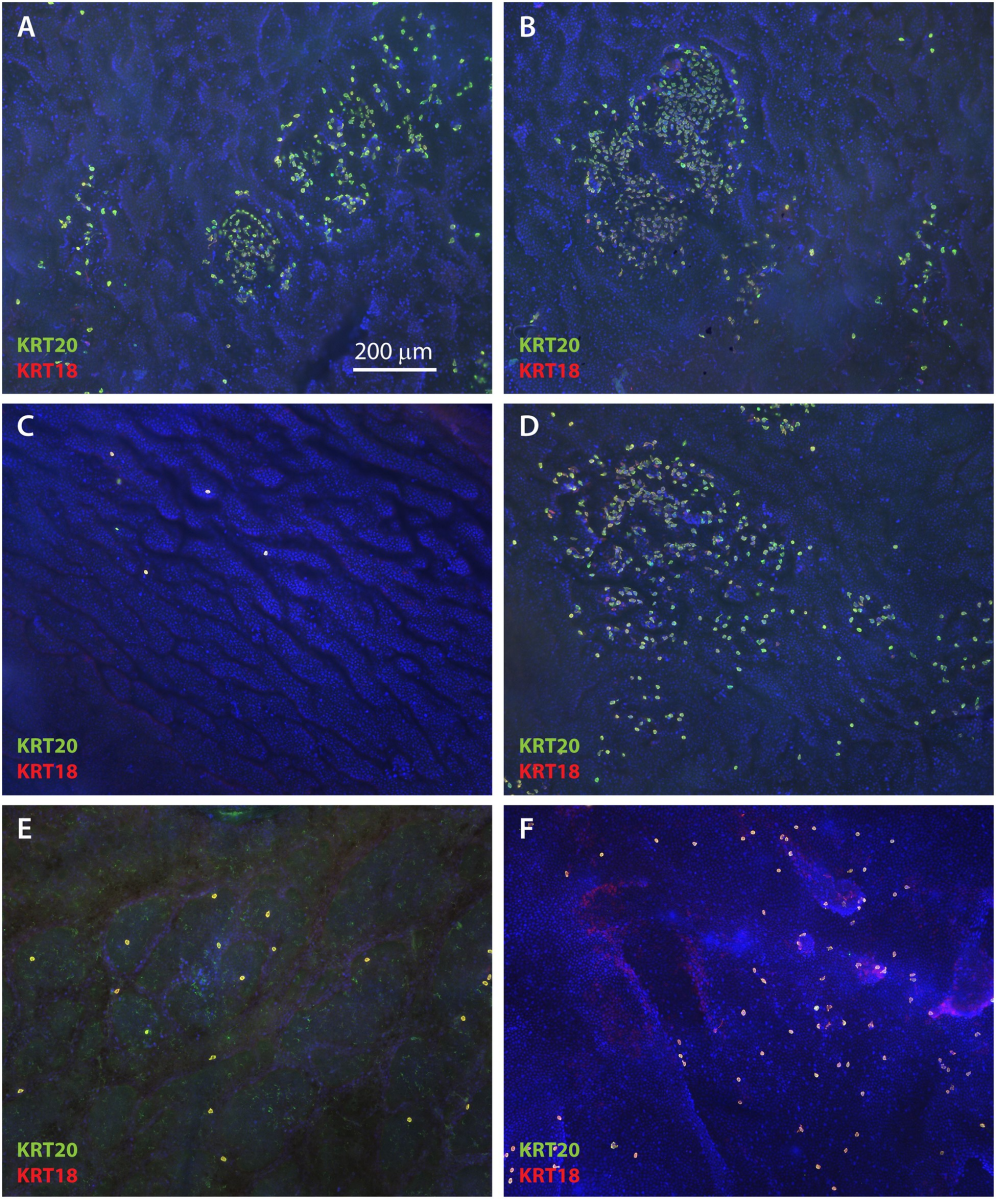
Merkel cell density in ESS is highly variable. Shown are *en face* images of epidermis from ESS at 12 weeks (**A-B**) and 14 weeks (**C-D**) after grafting, and normal human skin (**E-F**), immunostained with antibodies against KRT20 (green) and KRT18 (red); DAPI was used to counterstain nuclei (blue). Tissue was photographed at low magnification to illustrate variability in density of Merkel cells and relatively random distribution. Scale bar in **A** (200 μm) is same for all panels.

To better observe the spatial organization and density of Merkel cells in the basal layer of ESS, IHC was performed on epidermal sheets following enzymatic separation from the dermis. As observed in cross-sections, rare KRT20-positive cells were observed at 2 weeks after grafting (Fig 2A). At 4 weeks after grafting, small clusters of cells positive for KRT20 and KRT18 were observed (Fig 2B). These cells increased in frequency and were more widely dispersed from weeks six through 14 after grafting (Fig 2C–2G). As seen in cross sections of ESS *in vivo* (Fig 1A–1G), both oval-shaped and dendritic Merkel cells were observed (Fig 2). There did not appear to be any pattern to the distribution of Merkel cells in ESS; similar to the distribution of Merkel cells in normal human skin, the cells were found in clusters, linear patterns, or were present as widely dispersed cells (Figs 2 and 3). The density of cells was variable; this is illustrated in the lower magnification images of Merkel cells in epidermal sheets shown in Fig 3. Merkel cells appeared to be distributed randomly throughout the grafted ESS and were not more frequent in any particular region; i.e., cells were not more or less frequent at the wound margins compared with the centers of the grafts.

### Merkel cells in ESS are derived from grafted human epidermal cells

**Fig 4:**
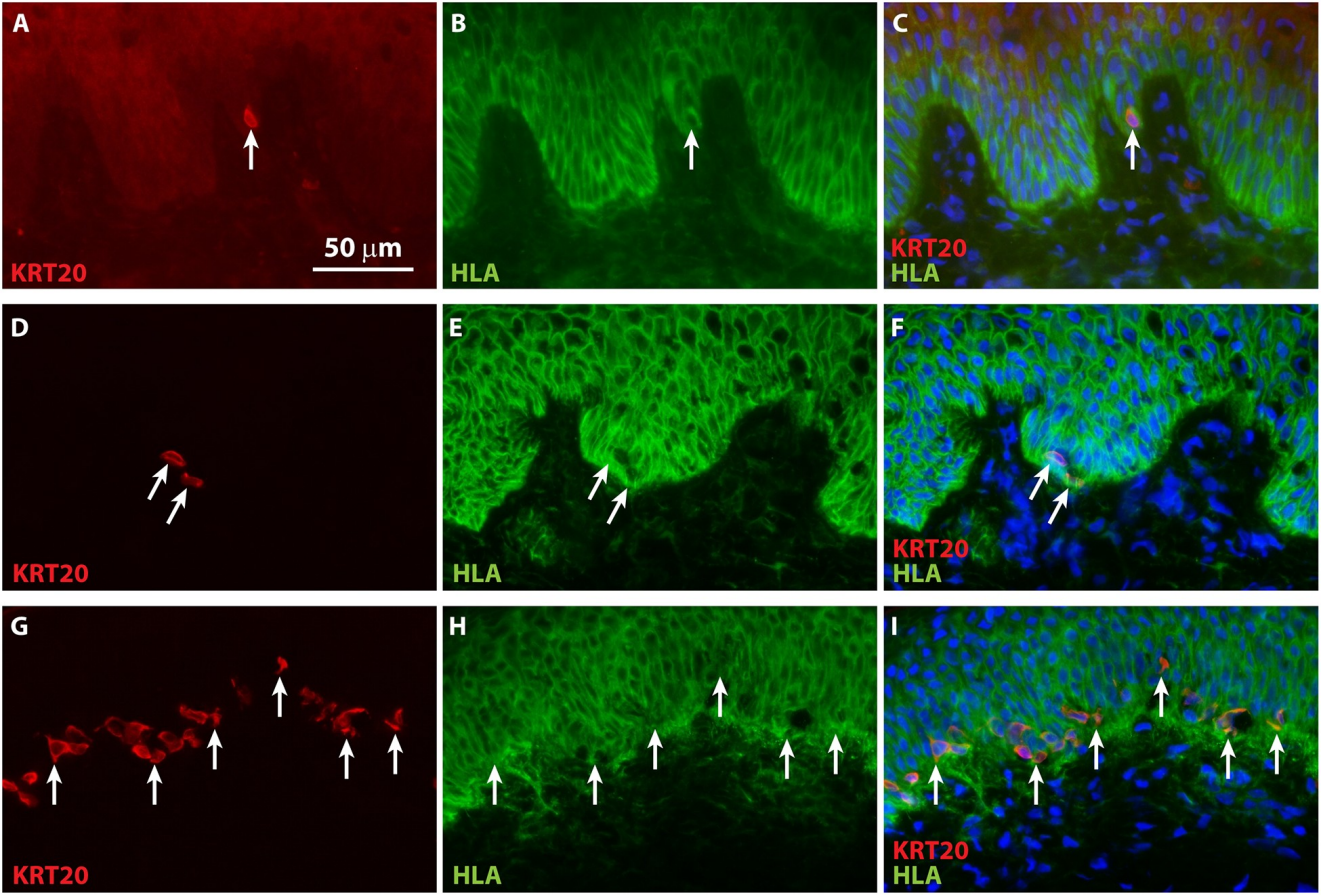
Demonstration of human cell engraftment in ESS *in vivo*. Immunohistochemistry was performed using an antibody against HLA-ABC (green) to localize human keratinocytes in ESS after grafting to mice. Merkel cells were visualized by staining with an antibody against KRT20 (red). Nuclei were counterstained using DAPI (blue; **C**, **F**, **I**). Sections are oriented with the epidermis at the top of each image. Note that images in each row depict a single section. **A-C**, ESS at 4 weeks after grafting. **D-F**, ESS at 8 weeks after grafting. **G-I**, ESS from 12 weeks after grafting. Arrows indicate examples of KRT20-positive Merkel cells in grafted human ESS. Scale bar in **A** is same for all sections (50 μm).

**Fig 5:**
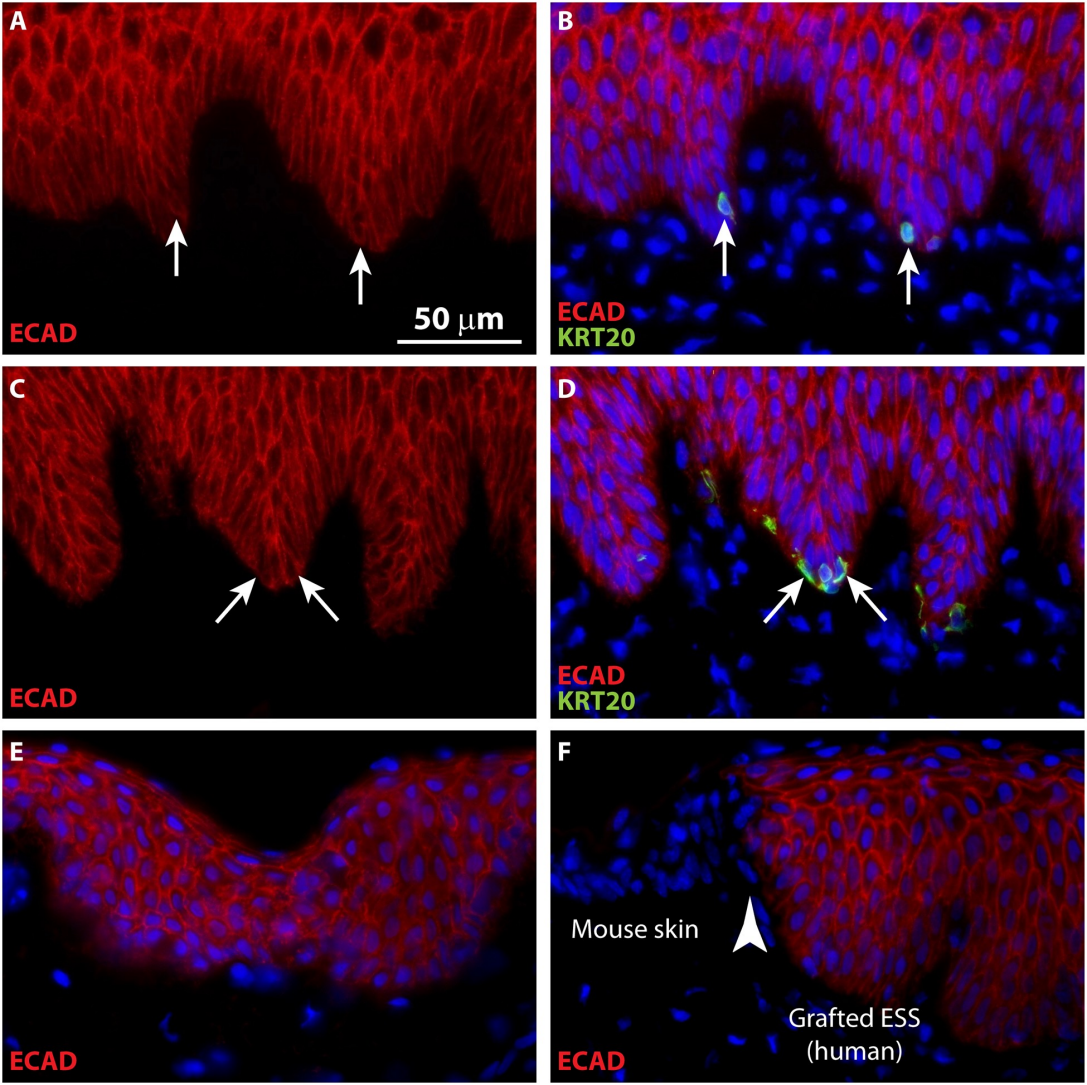
Merkel cells in grafted ESS are derived from human epidermal cells. Immunohistochemistry was performed using a human-specific antibody against E-cadherin (ECAD; red); Merkel cells were localized by KRT20 immunostaining (green). **A-B**, ESS at 6 weeks after grafting; **C-D**, ESS at 12 weeks after grafting. Sections are oriented with the epidermis at the top of each image; in **A**-**D**, each row depicts images of the same tissue section. Arrows depict examples of KRT20-positive Merkel cells in ESS. **E-F**, Controls for native human skin (**E**) and immunodeficient mouse skin (**F**) demonstrating specificity of anti-human E-cadherin antibody (red). Section shown in **F** depicts the border (arrowhead) between grafted human ESS (right) and flanking mouse skin (left). Nuclei were counterstained with DAPI (**B**, **D**, **E**, **F**; blue); scale bar in **A** is for all sections (50 μm).

To confirm engraftment of ESS *in vivo*, IHC was performed to localize human leukocyte antigen ABC (HLA-ABC) in grafted human cells. Co-localization of KRT20 and HLA in grafted ESS demonstrated the human origin of Merkel cells in ESS (Fig 4). Note that although the subcellular localization of KRT20 is distinct from HLA-ABC, which is expressed on the surface of the grafted human cells, these antigens are co-expressed in the same cells (Fig 4). To further confirm the human epidermal origin of the Merkel cells in ESS, IHC was performed using a species-specific antibody for human E-cadherin, an adhesion molecule expressed in epithelial cells (Fig 5). Specificity of the antibody was demonstrated by lack of staining of mouse epidermis flanking the grafted human ESS (Fig 5F). Co-localization of KRT20 and E-cadherin in ESS *in vivo* was observed (Fig 5A–5D), consistent with derivation of the Merkel cells from the grafted human keratinocytes.

**Fig 6:**
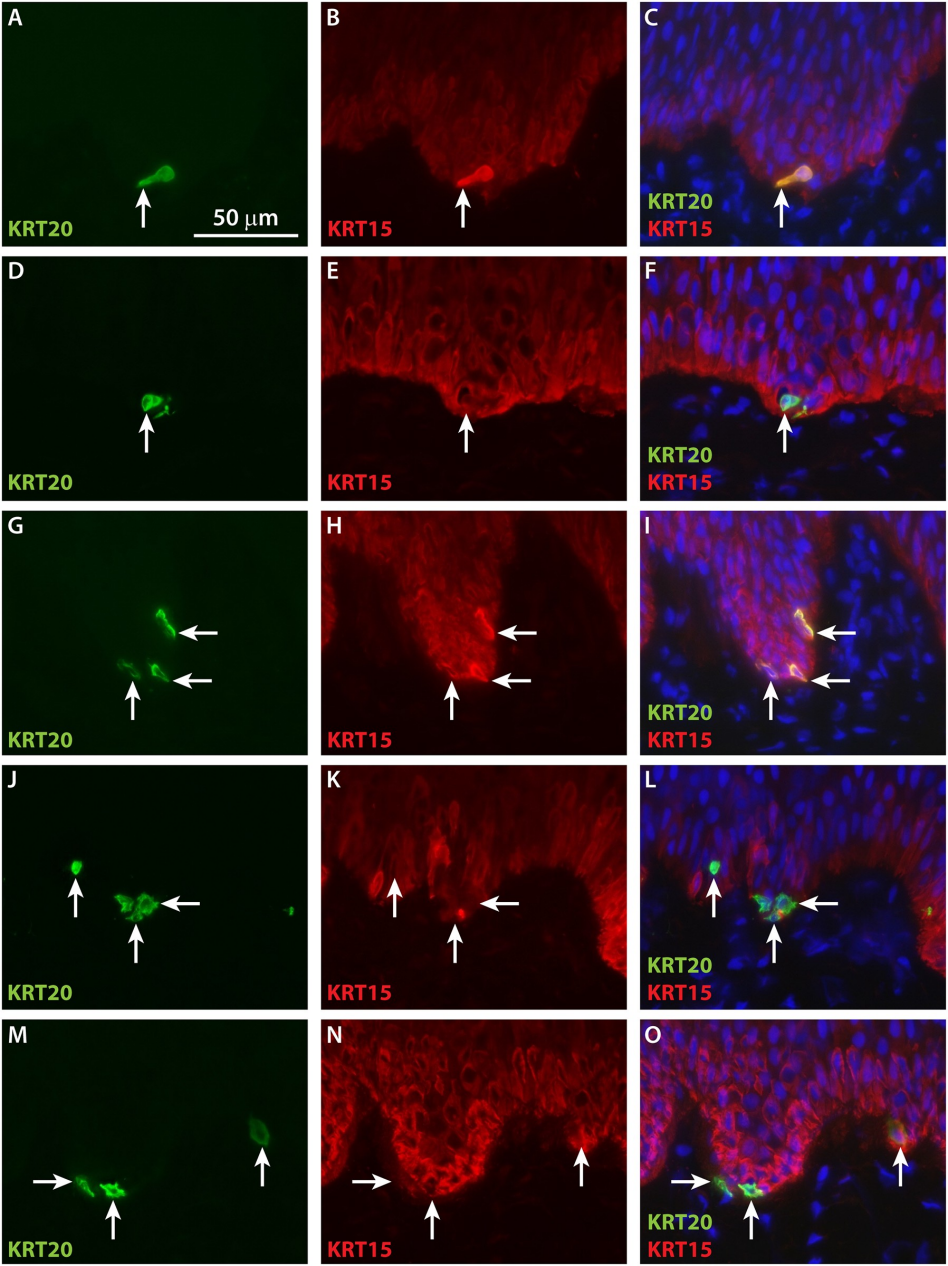
Co-localization of KRT20 and basal keratinocyte marker KRT15 in Merkel cells in grafted ESS. Immunohistochemistry was performed using antibodies against KRT20 (green) and KRT15 (red). Shown are sections of ESS excised from mice at 4 weeks (**A**-**C**), 6 weeks (**D**-**F**), 8 weeks (**G**-**I**), 12 weeks (**J**-**L**), and 14 weeks (**M**-**O**) after grafting. Sections are oriented with the epidermis at the top of each image. Each row of three images depicts the same section. Arrows indicate examples of KRT20-positive Merkel cells in grafted ESS. Nuclei were counterstained with DAPI (**C**, **F**, **I**, **O**; blue). Scale bar in **A** is for all sections (50 μm).

Localization of keratin 15 (KRT15), which is expressed in basal keratinocytes in human skin, was examined in grafted ESS. KRT15 was expressed diffusely in basal keratinocytes by four weeks *in vivo*. In contrast, bright KRT15 staining of Merkel cells was observed at four weeks, and the subcellular localization pattern of this staining overlapped that of KRT20 (Fig 6A–6C). At later time points, co-localization of KRT15 and KRT20 was observed in the same basal cells; although most Merkel cells displayed perinuclear KRT20 staining and diffuse cytoplasmic KRT15 staining, sporadic Merkel cells were observed that also displayed perinuclear KRT15 staining (e.g., Fig 6G–6I).

Keratin 19 (KRT19) has been considered a marker for hair follicle stem cells in humans and mice, but in interfollicular epidermis of hairy skin its expression is reportedly limited to Merkel cells [47]. Co-localization of KRT19 and KRT20 was observed in both sections of ESS (S4 Fig) and in epithelial sheets of ESS viewed *en face* (S5 Fig). Occasional KRT20-positive cells were observed that did not express KRT19, but all KRT19 cells in ESS *in vivo* co-expressed KRT20 (S4 and S5 Figs). Co-localization of KRT19 and KRT20 was also observed in normal human skin (S5 Fig).

### Merkel cells in grafted ESS express neuroendocrine markers and are associated with neurons

**Fig 7:**
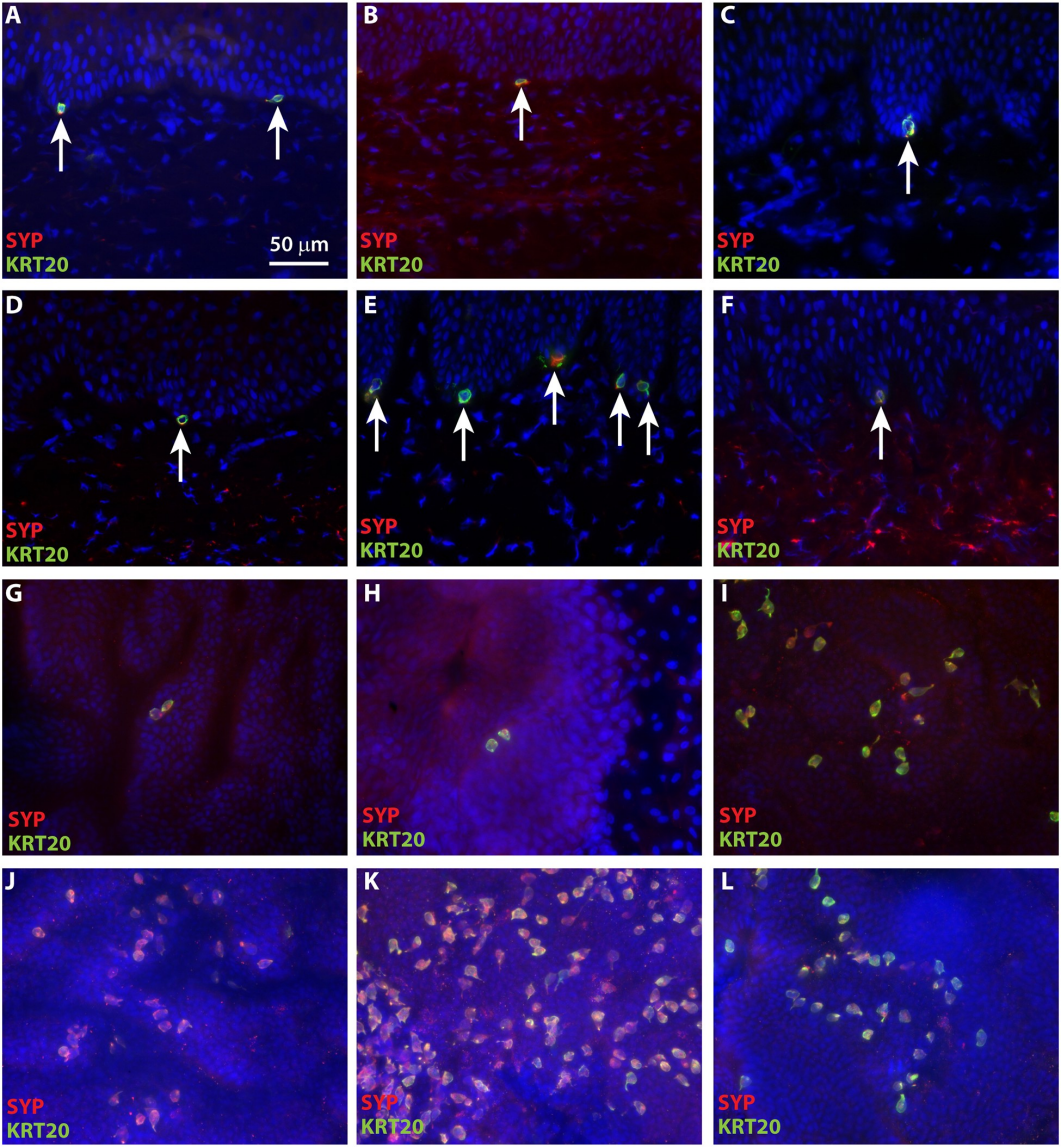
Co-localization of KRT20 and neuroendocrine marker synaptophysin (SYP) in grafted ESS. Immunohistochemistry was performed using antibodies against KRT20 (green) and SYP (red); nuclei were counterstained with DAPI (blue). **A**-**F**, Cross sections of ESS at 4 weeks (**A**), 6 weeks (**B**), 8 weeks (**C**), 10 weeks (**D**), 12 weeks (**E**), and 14 weeks (**F**) after grafting; sections are oriented with the epidermis at the top of each image. Arrows indicate examples of KRT20/SYP-positive Merkel cells. **G**-**L**, Epidermal sheets of ESS at 4 weeks (**G**), 6 weeks (**H**), 8 weeks (**I**), 10 weeks (**J**), 12 weeks (**K**), and 14 weeks (**L**) after grafting, photographed *en face* following immunostaining. Scale bar in **A** is for all images (50 μm).

Synaptophysin (SYP), an integral membrane protein found in presynaptic vesicles, is considered a marker for neuroendocrine cells [48, 49]. Consistent with their neuroendocrine function in skin, human Merkel cells have been reported to express SYP [50]. We observed co-localization of KRT20 and SYP in ESS as early as four weeks after grafting (Fig 7). In addition, chromogranin A (CGA), which is also expressed cells of the neuroendocrine system [51], was expressed in KRT20-positive cells in ESS (S6 Fig), consistent with the identification of these cells as Merkel cells.

**Fig 8:**
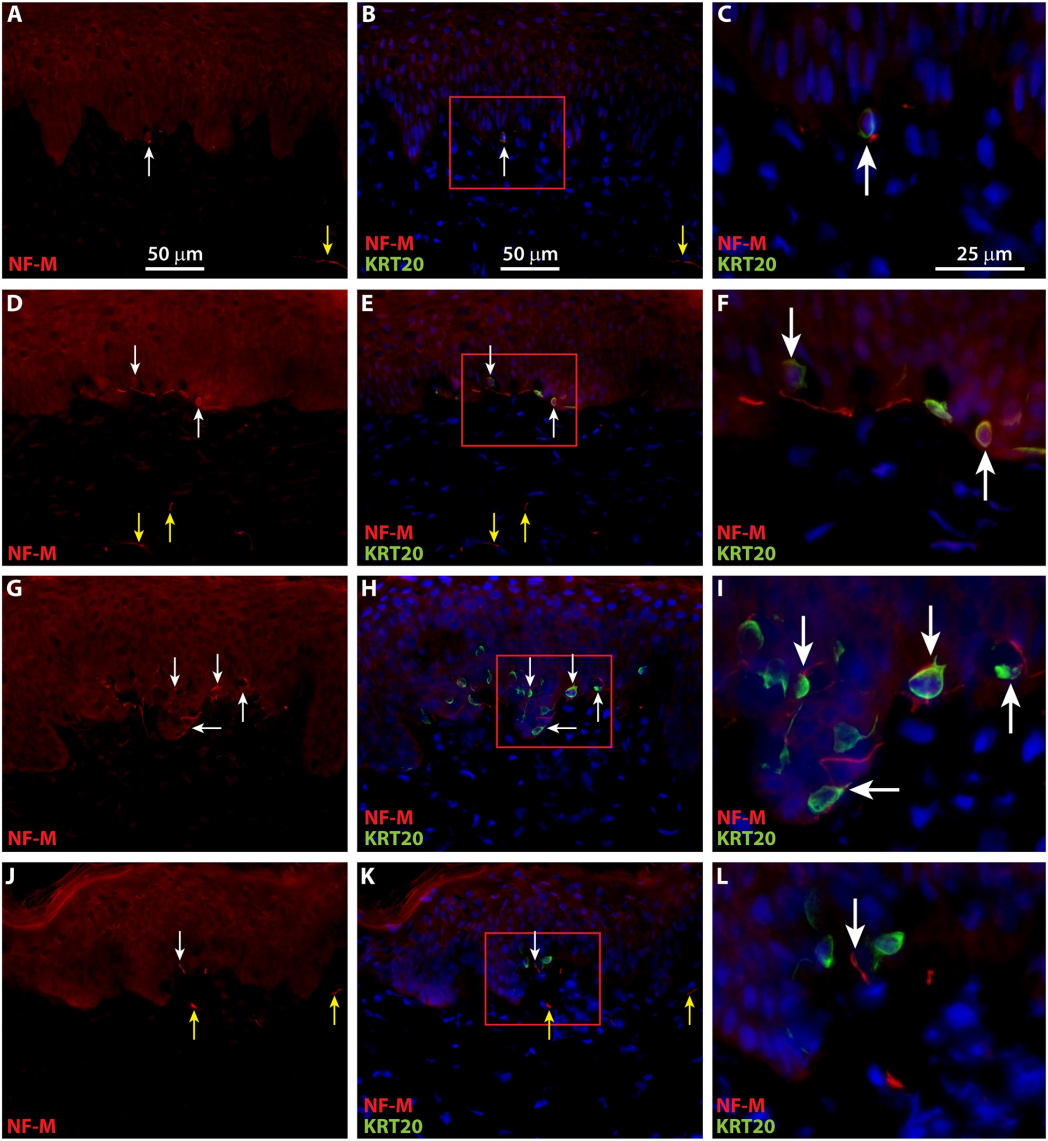
Merkel cells in grafted ESS are associated with neurons expressing neurofilament medium (NF-M). Immunochemistry with antibodies against NF-M (red) and KRT20 (green) was used to localize neurons and Merkel cells, respectively, in ESS after grafting to mice. Nuclei were counterstained with DAPI (blue; **B**, **C**, **E**, **F**, **H**, **I**, **K**, **L**). Shown are cross sections of ESS at 4 weeks (**A**-**C**), 6 weeks (**D**-**F**), 8 weeks (**G**-**I**), and 12 weeks (**J**-**L**) after grafting; each row contains images of the same section. Images shown in the right-hand column (**C**, **F**, **I**, L) are 3-fold magnified images of boxed areas in center column (**B**, **E**, **H**, **K**). Scale bars in panels in first row are same for all images in column. White arrows indicate examples of NF-M-positive nerves associated with or in proximity to Merkel cells; yellow arrows indicate NF-M-positive nerves not associated with Merkel cells.

**Fig 9:**
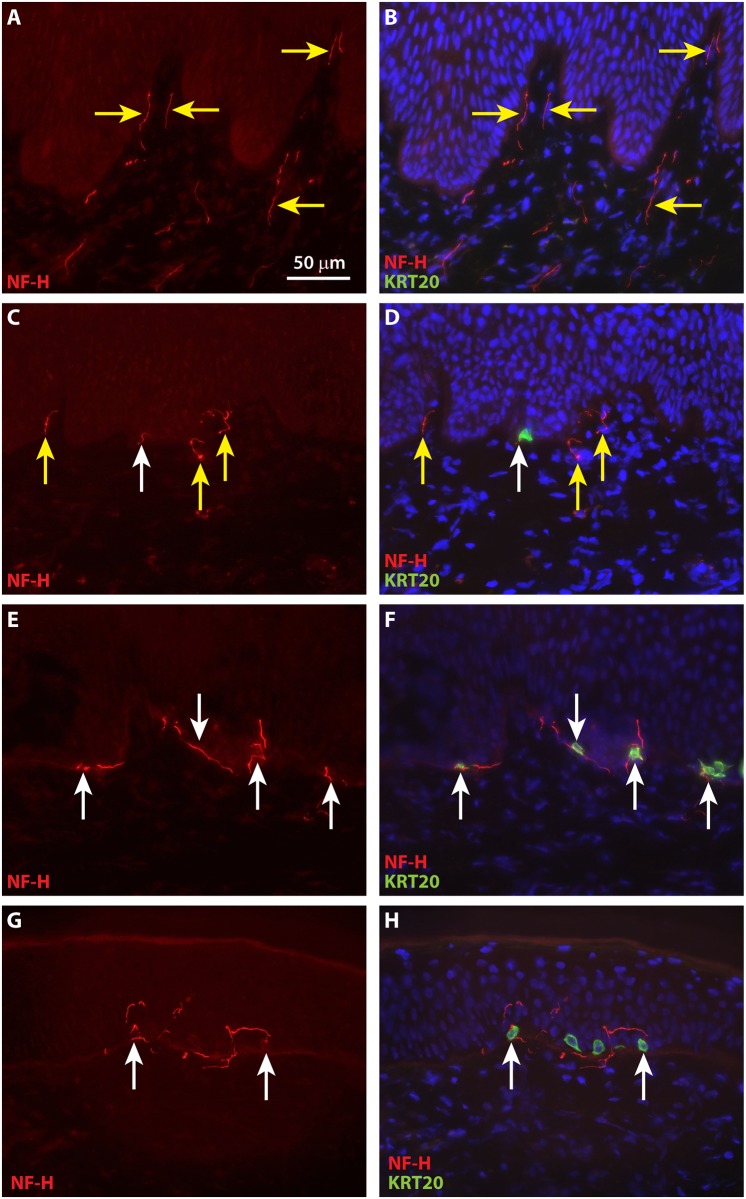
Merkel cells in grafted ESS are associated with neurons expression neurofilament heavy (NF-M) by eight weeks after grafting. Immunochemistry with antibodies against NF-H (red) and KRT20 (green) was used to localize neurons and Merkel cells, respectively, in ESS after grafting to mice. Nuclei were counterstained with DAPI (blue; **B**, **D**, **F**, **H**). Shown are cross sections of ESS at 4 weeks (**A**-**B**), 8 weeks (**C**-**D**), 12 weeks (**E**-**F**), and 14 weeks (**G**-**H**) after grafting; each row contains images of the same section. Scale bars in **A** is same for all images. White arrows indicate examples of NF-H-positive nerves associated with or in proximity to Merkel cells; yellow arrows indicate NF-H-positive nerves not associated with Merkel cells.

To localize neurons in ESS *in vivo*, IHC was performed using antibodies against neurofilament medium (NF-M) and neurofilament heavy (NF-H). NF-M and NF-H are both expressed in neurons in the central and peripheral nervous systems. Developmentally, NF-M expression precedes that of NF-H, which appears later during axonal maturation, concomitant with myelination [52, 53]. Expression of NF-H has been demonstrated previously in myelinated afferents associated with touch domes of Merkel cells in mice [29]. Small NF-M-positive nerve fibers were observed in ESS as early as four weeks after grafting, and infrequently these were co-localized with KRT20-positive Merkel cells (Fig 8A–8C). NF-M-positive afferents associated with Merkel cells were observed at higher frequencies at later time points after grafting (Fig 8D–8L). Although NF-H positive neurons were observed in the upper dermis of ESS at 4 weeks after grafting, they were not found in association with Merkel cells until 8 weeks after grafting (Fig 9A–9D). These Merkel cell-neurite complexes were observed more frequently by 12 to 14 weeks after grafting (Fig 9E–9H).

### Detection of Merkel cells in primary keratinocyte cultures

To determine whether Merkel cells derived from the human skin samples may have persisted in the primary keratinocyte cultures used for preparation of ESS, keratinocytes of the same donor strain were recovered from cryopreservation and expanded in culture at the same ratios as those used for ESS preparation. FACS analysis indicated that 0.14% of the cells in the primary cell population at first passage were KRT20-positive, and 0.31% of the cells were KRT20-positive at the second passage. To determine whether the presence of KRT20-positive cells was specific for primary cultures of a single patient, FACS analysis was performed using primary keratinocytes isolated from a second, unrelated donor. That analysis identified 0.23% of the cell population as KRT20-positive at first passage, and 0.10% KRT20-positive at second passage. Statistical analysis indicated that the difference in the percentage of KRT20-positive cells in first passage and second passage cultures was not statistically significant (p = 0.9156), suggesting that the proportion of cells positive for KRT20 does not change significantly during passage in culture.

## Discussion

Loss of sensation is commonly reported by burn survivors and can persist for decades after burn injury [11–15]. Although deficits in innervation have been reported, relatively little is known about recovery of Merkel cell populations in burn scars. In one study from 1990, Merkel cells were observed in healed autograft but they were not associated with nerves [35]; in a 1994 study, Merkel cells were observed to be present only infrequently in healed skin grafts [34]. In mice, deletion of Piezo2 channels in Merkel cells was associated with increased pain sensitivity, in addition to reduced fine touch sensation, and absence of Merkel cells in mouse skin caused increased itching [30, 31]. It is not yet known whether Merkel cells have similar roles in pain and itch in human skin. If they do, it is possible that the absence of Merkel cell-neurite complexes in burn scars and skin grafts may contribute to not only the decreased fine touch sensation, but also the increased pain and itch experienced by burn survivors. Thus, the presence of Merkel cells in healed ESS would have important implications for functional recovery after burn injury. Hypothetically, Merkel cells in ESS may contribute to restoration of fine touch sensation while reducing pain and itch.

Although Merkel cells are known to express cytokeratins 8, 18, 19, and 20, KRT20 is considered a general marker for Merkel cells [32, 40]. KRT20 is expressed in epithelium of the gastrointestinal system and bladder, and in tumors of these tissues, but in skin its expression is limited to Merkel cells and Merkel cell carcinoma [32, 46]. Expression of neuroendocrine markers SYP and CGA has also been reported in Merkel cells [48–51]. The co-expression of KRT20, KRT18, KRT19, SYP, and CGA in cells in the basal epidermis of ESS after grafting, and the association of these cells with nerves, strongly suggests that these cells are Merkel cells. Expression of human E-cadherin and HLA-ABC in the Merkel cells observed in ESS indicates that these cells are derived from grafted human cells.

It is unclear whether the Merkel cells observed in ESS *in vivo* were derived from Merkel cells that originated from human skin and were passively transferred and/or proliferated during *in vitro* primary culture of epidermal cells, or whether these cells differentiated from progenitor cells present in the primary culture. Persistence of KRT20-positive Merkel cells at low proportions in primary epidermal cultures has been previously reported; however, in the majority of cultures, those cells decreased, rather than increased, in number after sequential passaging *in vitro* [54]. Presumably, the low percentage of epidermal cells identified in our primary cultures that stained positive for KRT20 are Merkel cells; however, we cannot rule out the possibility that these cells represent a population of keratinocytes expressing a de-differentiated phenotype. Other investigators have reported nonspecific expression of simple keratins in up to 20% of primary epidermal cells [35]. If the KRT20-positive primary cells *in vitro* are Merkel cells, they appear to be either post-mitotic or to have very low proliferation rates in culture. Although a statistically significant increase was not demonstrated, their numbers may have increased two-fold in culture during a period in which keratinocytes underwent approximately five population doublings (~32-fold expansion in cell number). No KRT20-positive epidermal cells were detected in ESS at any time point examined *in vitro*. This suggests that the KRT20-positive Merkel cells observed *in vivo* were not derived from cells passively transferred during *in vitro* culture; however, it remains possible that Merkel cells were present in ESS *in vitro* at numbers below the limits of detection.

Merkel cells in human skin have been described as post-mitotic because dividing Merkel cells have never been detected [55]. Thus, it was suggested that Merkel cells in human skin might be renewed from a population of undifferentiated keratinocytes or common progenitor cells [55]. Labeling experiments in mice have shown that Merkel cells are long-lived cells that are produced very rarely in adult skin under homeostatic conditions [56]. However, upon mild skin injury, increased numbers of Merkel cells were produced, and fate-mapping showed that their differentiation was induced from touch dome keratinocytes [56]. Other studies in mice suggested that during development, Merkel cells originate from epidermal progenitors [57, 58]. In human skin, expression of multiple markers of the epidermal lineage in Merkel cells was described, consistent with their origination from epidermal progenitors [59]. Previous gene expression profiling studies revealed that after grafting, ESS exhibit a hyperproliferative phenotype resembling a wound healing response [60]. Hypothetically, this wound healing phenotype may stimulate differentiation and proliferation of Merkel cells derived from epidermal progenitors in ESS. This may explain the appearance of Merkel cells in ESS *in vivo* and their dramatic increase in number over time after grafting, and is consistent with previous studies describing the epidermal origin of Merkel cells. Although we are currently unable to demonstrate conclusively that Merkel cells in ESS are derived from epidermal progenitors rather than Merkel cells passively transferred from human skin, the dramatic increase in their numbers after grafting strongly suggests that Merkel cells proliferate in ESS *in vivo*.

Qualitative analysis of innervation suggests that Merkel cells in ESS arise and expand in number prior to interactions with nerves from the mouse host. Rare, widely separated Merkel cells were observed as early as 2 weeks after grafting, but were not associated with neurons at this time point. Sensory afferents expressing NF-M were observed in association with Merkel cells by 4 weeks after grafting. Afferents expressing NF-H, which is reportedly found in more mature, myelinated neurons, were observed in the dermis of ESS at 4 weeks after grafting, but were not found in association with Merkel cells until 8 weeks after grafting. As previously observed in rodent and human skin [26, 43, 45], Merkel cells in ESS were heterogeneous in shape, displaying either a dendritic phenotype or more oval shape. It has been reported that in rodents, oval shaped Merkel cells tend to be innervated whereas dendritic cells may not be associated with nerves [43]. Although we observed a high proportion of dendritic-shaped Merkel cells in ESS, particularly at weeks 4–6 after grafting (Figs 1 and 2), we are currently unable to determine whether there is a correlation between Merkel cell shape and innervation.

## Conclusion

ESS were shown to provide stable wound closure of excised burn wounds in pediatric patients, but innervation status of healed ESS has not been previously examined. Innervation has been studied preclinically in other engineered skin models. For example, a collagen-chitosan scaffold populated with fibroblasts and keratinocytes was found to be innervated 60 days after transplantation to mice, and incorporation of Schwann cells *in vitro* accelerated innervation after grafting [61, 62]. Merkel cells were not described in either of those engineered skin models. The co-localization of Merkel cell markers and neuroendocrine proteins in cells of the basal epidermis of ESS *in vivo* strongly suggests that these cells are Merkel cells. The current study indicates that innervation of ESS is initiated within four weeks after transplantation, and by four to 6 weeks after grafting, neurons are found in association with Merkel cells. This suggests formation of Merkel cell-neurite complexes in ESS *in vivo*, which to our knowledge has not been previously demonstrated in a tissue-engineered skin substitute. This observation suggests that fine touch sensation may be restored in healed engineered skin; however, functional studies will be required to confirm the role of Merkel cells in recovery of touch sensation after grafting.

## Supporting information

S1 Fig Histological sections of engineered skin substitutes (ESS). Shown are hematoxylin & eosin (H&E) stained sections of ESS from day 10 *in vitro* (A), prior to grafting, and from week 2 (B), week 4 (C), week 6 (D), week 8 (E), week 10 (F), week 12 (G), and week 14 (H) after grafting to mice. Arrows in panels A and B indicate examples of the dense reticulations of the bovine collagen scaffold; these are observed *in vitro* but are less frequent after grafting and are rarely observed after 10 weeks *in vivo*. Newly synthesized human collagen, which appears light pink in H&E-stained sections, is indicated by asterisks in panel B. Scale bar in A is for all sections (200 μm).
(PDF)

S2 Fig Mice grafted with engineered skin substitutes (ESS). Shown are photographs of grafted mice from week 2 (A), week 4 (B), week 6 (C), week 8 (D), week 10 (E), week 12 (F), and week 14 (G) after grafting. Arrows indicate corners of grafted ESS. Note that different mice are shown in each panel; photographs were taken immediately before euthanasia and biopsy collection. Spots of pigment observed in some grafts are due to passenger melanocytes present non-specifically in the epidermal cultures used for preparation of ESS.
(PDF)

S3 Fig Identification of Merkel cells in human engineered skin substitutes (ESS) 15 weeks after grafting to immunodeficient mice. ESS were prepared with cells of a 33-year-old donor and, separately, a 15-year-old donor; representative images are shown. **A**, Localization of KRT20 (green) and KRT18 (red) in Merkel cells (arrows) in ESS 15 weeks after grafting. DAPI was used to counterstain nuclei (blue); all panels in **A** depict images of the same section. The dermal-epidermal junction is indicated by the dashed white line. **B**, Engraftment of human cells in ESS was confirmed by immunohistochemistry with anti-HLA-ABC antibody (green). KRT20 (red) is localized to Merkel cells that are HLA-ABC-positive (arrows), confirming their human origin. DAPI was used to counterstain nuclei (blue); all panels in **B** depict images of the same section.
(PDF)

S4 Fig Co-localization of KRT19 and KRT20 in Merkel cells in sections of ESS *in vivo*. Shown are sections of ESS excised from mice at 4 weeks (**A**-**C**), 6 weeks (**D**-**F**), 8 weeks (**G**-**I**), and 12 weeks (**J**-**L**) after grafting to mice. Immunohistochemistry was performed using antibodies against KRT19 (red) and KRT20 (green); DAPI was used to counterstain nuclei (**C**, **F**, **I**, **L**; blue). Each row depicts images of a single representative section. White arrows indicate examples of cells staining positive for both KRT19 and KRT20; yellow arrows indicate KRT20-positive cells that do not appear to express KRT19. Scale bar in **A** is for all sections (50 μm).
(PDF)

S5 Fig Co-localization of KRT19 and KRT20 in Merkel cells in ESS epidermal sheets. Shown are epidermal sheets of ESS excised from mice at 2 weeks (**A**-**C**), 8 weeks (**D**-**F**), and 14 weeks (**G**-**I**), and epidermal sheets of normal human skin (**J**-**L**), stained using antibodies against KRT19 (red) and KRT20 (green); DAPI was used to counterstain nuclei (**C**, **F**, **I**, **L**; blue). Each row depicts three images of the same representative microscopic field. White arrows indicate examples of cells staining positive for both KRT19 and KRT20; yellow arrows indicate KRT20-positive cells that do not appear to express KRT19. Scale bar in **A** is for all sections (50 μm).
(PDF)

S6 Fig Expression of neuroendocrine marker chromogranin A (CGA) in engineered skin *in vivo*. Shown are *en face* images of ESS collected at 14 weeks after grafting. Arrows indicate examples of cells expressing both KRT20 (green) and CGA (red); DAPI (blue) was used to counterstain nuclei.
(PDF)
