# Supplementary material for: Identification of Merkel cells associated with neurons in engineered skin substitutes after grafting to full thickness wounds
Source: PLoS One. 2019 Mar 5;14(3):e0213325. doi: 10.1371/journal.pone.0213325 (PMC6400390; doi:10.1371/journal.pone.0213325)
Supplement: S1 Fig — Shown are hematoxylin & eosin (H&E) stained sections of ESS from day 10 in vitro (A), prior to grafting, and from week 2 (B), week 4 (C), week 6 (D), week 8 (E), week 10 (F), week 12 (G), and week 14 (H) after grafting to mice. Arrows in panels A and B indicate examples of the dense reticulations of the bovine collagen scaffold; these are observed in vitro but are less frequent after grafting and are rarely observed after 10 weeks in vivo. Newly synthesized human collagen, which appears light pink in H&E-stained sections, is indicated by asterisks in panel B. Scale bar in A is for all sections (200 μm). (PDF) [file pone.0213325.s001.pdf]

Supporting Information: S1 Figure

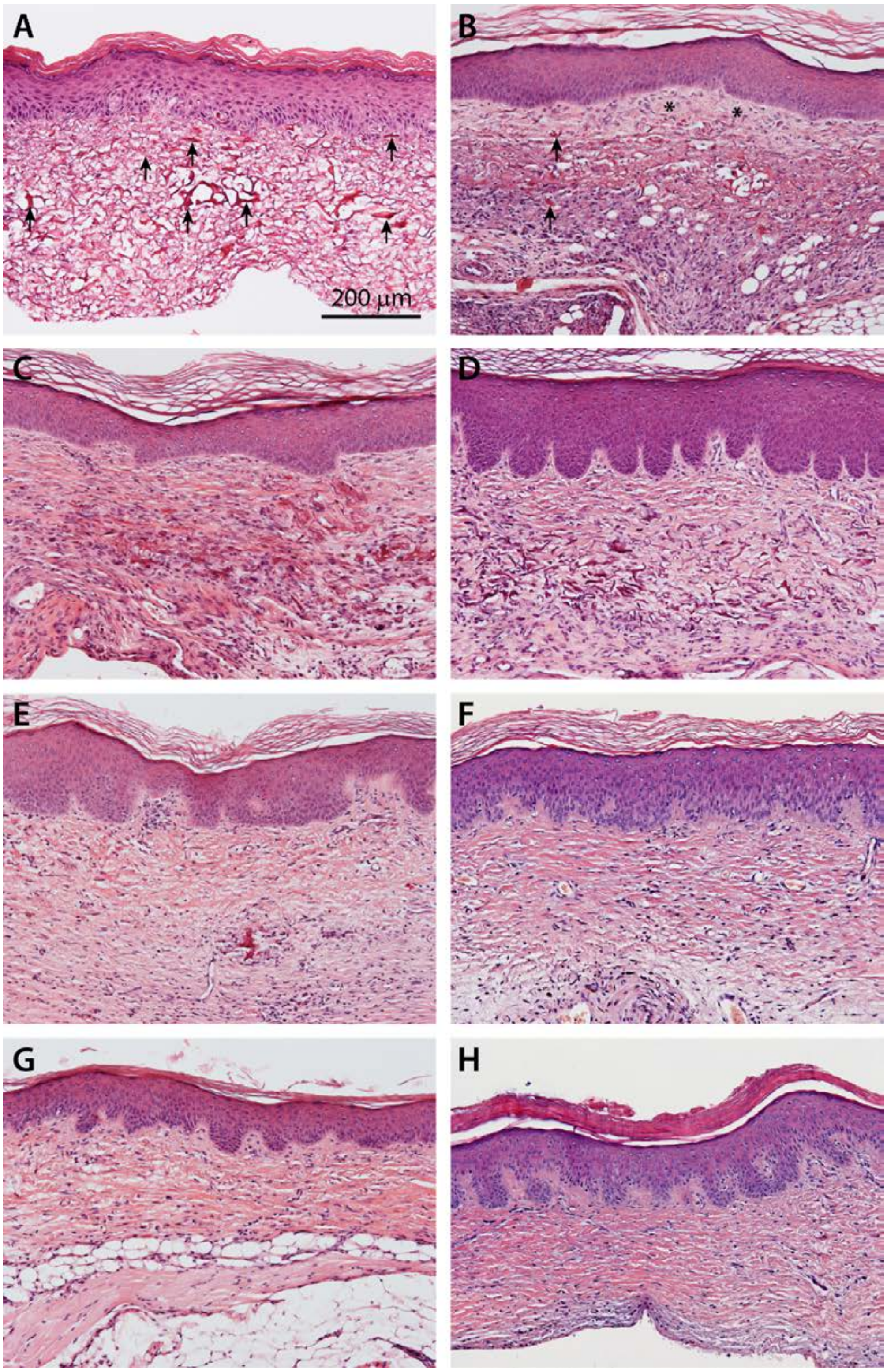

S1. Histological sections of engineered skin substitutes (ESS). Shown are hematoxylin & eosin (H&E) stained sections of ESS from day 10 *in vitro* (A), prior to grafting, and from week 2 (B), week 4 (C), week 6 (D), week 8 (E), week 10 (F), week 12 (G), and week 14 (H) after grafting to mice. Arrows in panels A and B indicate examples of the dense reticulations of the bovine collagen scaffold; these are observed *in vitro* but are less frequent after grafting and are rarely observed after 10 weeks *in vivo*. Newly synthesized human collagen, which appears light pink in H&E-stained sections, is indicated by asterisks in panel B. Scale bar in A is for all sections (200 µm).
