# Supplementary material for: Identification of Merkel cells associated with neurons in engineered skin substitutes after grafting to full thickness wounds
Source: PLoS One. 2019 Mar 5;14(3):e0213325. doi: 10.1371/journal.pone.0213325 (PMC6400390; doi:10.1371/journal.pone.0213325)
Supplement: S2 Fig — Shown are photographs of grafted mice from week 2 (A), week 4 (B), week 6 (C), week 8 (D), week 10 (E), week 12 (F), and week 14 (G) after grafting. Arrows indicate corners of grafted ESS. Note that different mice are shown in each panel; photographs were taken immediately before euthanasia and biopsy collection. Spots of pigment observed in some grafts are due to passenger melanocytes present non-specifically in the epidermal cultures used for preparation of ESS. (PDF) [file pone.0213325.s002.pdf]

Supporting Information: S2 Figure

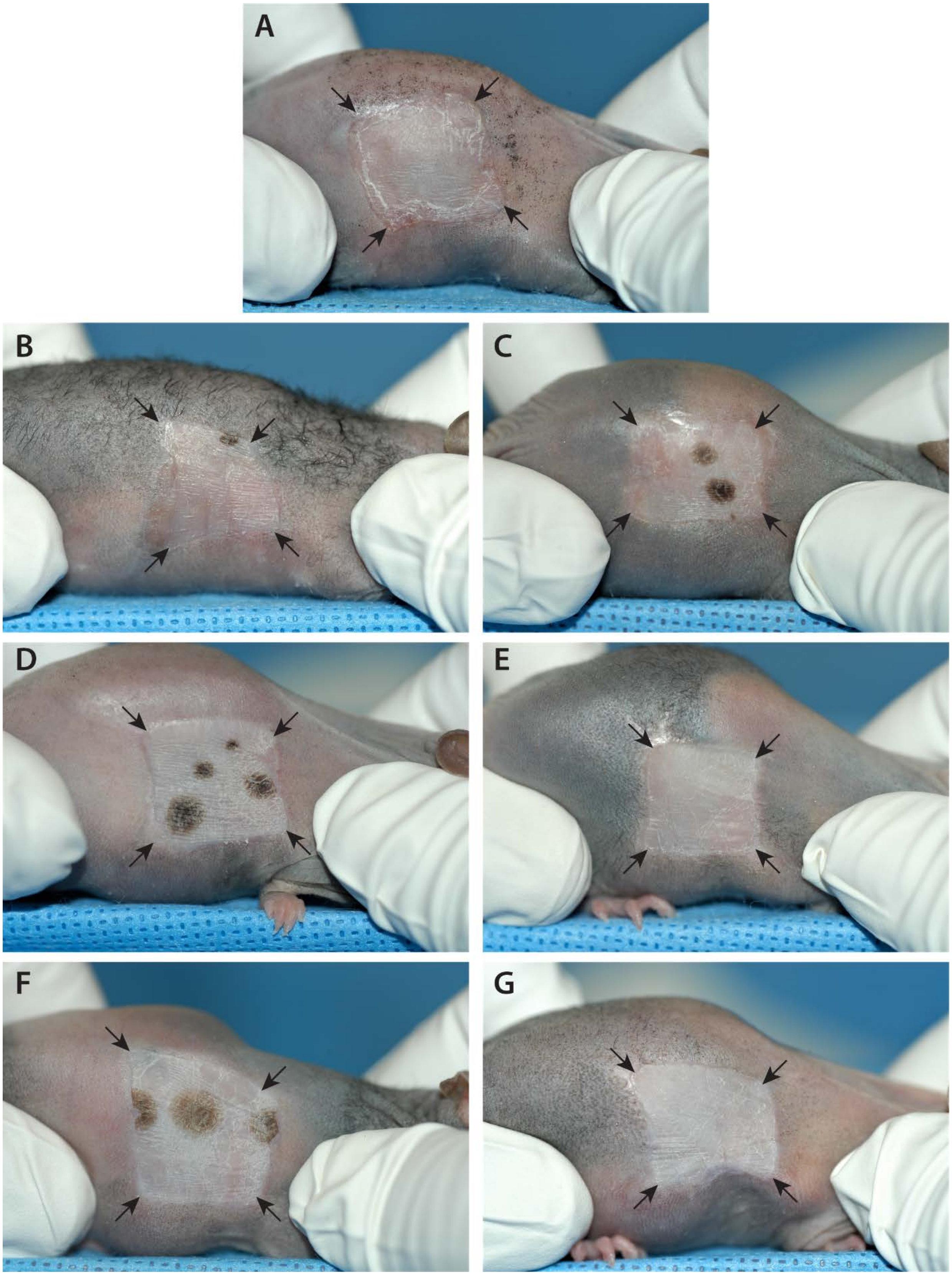

S2. Mice grafted with engineered skin substitutes (ESS). Shown are photographs of grafted mice from week 2 (A), week 4 (B), week 6 (C), week 8 (D), week 10 (E), week 12 (F), and week 14 (G) after grafting. Arrows indicate corners of grafted ESS. Note that different mice are shown in each panel; photographs were taken immediately before euthanasia and biopsy collection. Spots of pigment observed in some grafts are due to passenger melanocytes present non-specifically in the epidermal cultures used for preparation of ESS.
