# Supplementary material for: Identification of Merkel cells associated with neurons in engineered skin substitutes after grafting to full thickness wounds
Source: PLoS One. 2019 Mar 5;14(3):e0213325. doi: 10.1371/journal.pone.0213325 (PMC6400390; doi:10.1371/journal.pone.0213325)
Supplement: S3 Fig — ESS were prepared with cells of a 33-year-old donor and, separately, a 15-year-old donor; representative images are shown. A, Localization of KRT20 (green) and KRT18 (red) in Merkel cells (arrows) in ESS 15 weeks after grafting. DAPI was used to counterstain nuclei (blue); all panels in A depict images of the same section. The dermal-epidermal junction is indicated by the dashed white line. B, Engraftment of human cells in ESS was confirmed by immunohistochemistry with anti-HLA-ABC antibody (green). KRT20 (red) is localized to Merkel cells that are HLA-ABC-positive (arrows), confirming their human origin. DAPI was used to counterstain nuclei (blue); all panels in B depict images of the same section. (PDF) [file pone.0213325.s003.pdf]

Supporting Information: S3 Figure

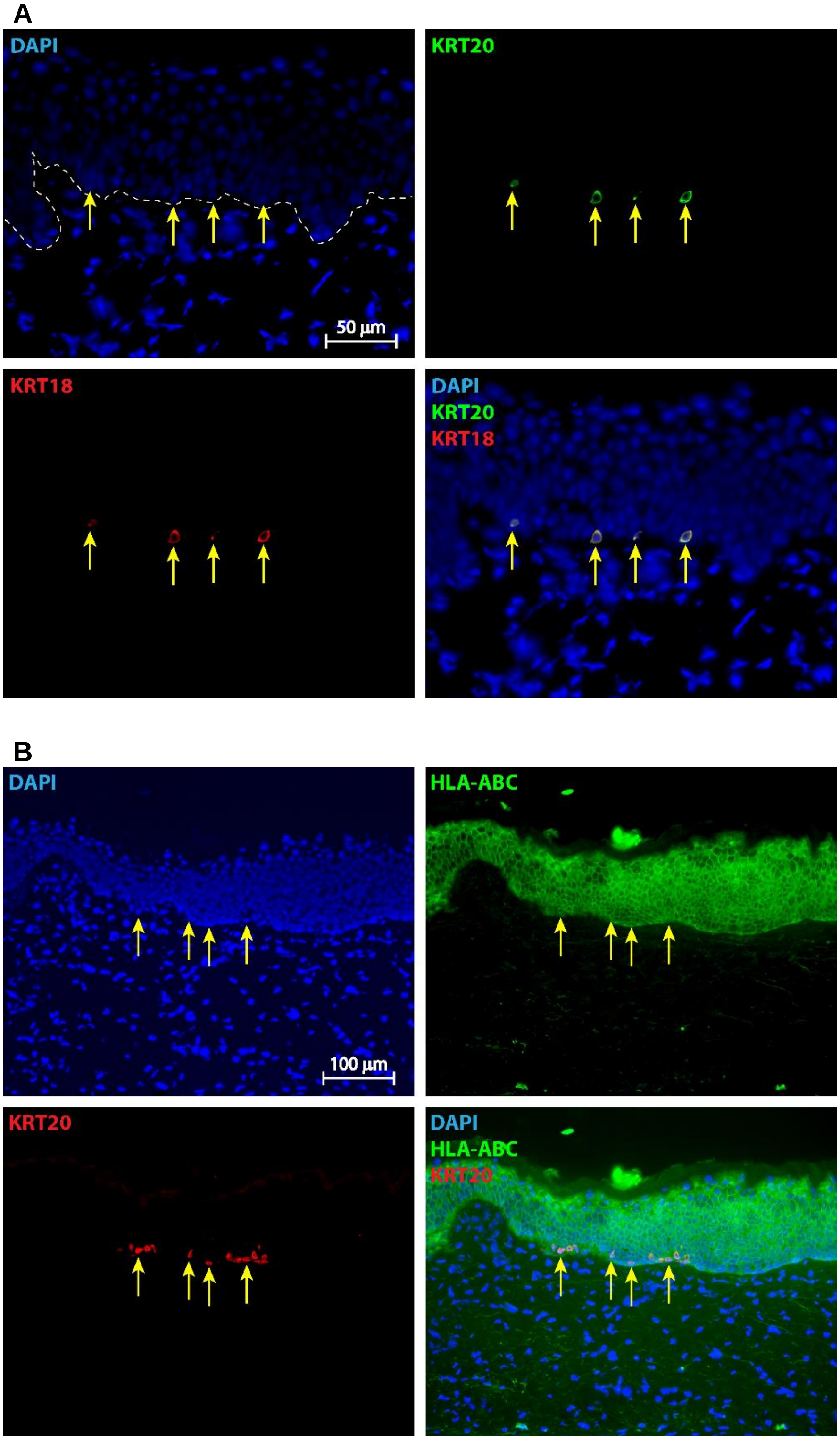

S3. Identification of Merkel cells in human engineered skin substitutes (ESS) 15 weeks after grafting to immunodeficient mice. ESS prepared with cells of a 33 year old female donor are shown; similar results were obtained using cells of a 15 year old male donor (not shown). **A**, Localization of KRT20 (green) and KRT18 (red) in Merkel cells (arrows) in ESS 15 weeks after grafting. DAPI was used to counterstain nuclei (blue); all panels in **A** depict images of the same section. The dermal-epidermal junction is indicated by the dashed white line. **B**, Engraftment of human cells in ESS was confirmed by immunohistochemistry with anti-HLA-ABC antibody (green). KRT20 (red) is localized to Merkel cells that are HLA-ABC-positive (arrows), confirming their human origin. DAPI was used to counterstain nuclei (blue); all panels in **B** depict images of the same section.
