# Supplementary material for: Identification of Merkel cells associated with neurons in engineered skin substitutes after grafting to full thickness wounds
Source: PLoS One. 2019 Mar 5;14(3):e0213325. doi: 10.1371/journal.pone.0213325 (PMC6400390; doi:10.1371/journal.pone.0213325)
Supplement: S4 Fig — Shown are sections of ESS excised from mice at 4 weeks (A-C), 6 weeks (D-F), 8 weeks (G-I), and 12 weeks (J-L) after grafting to mice. Immunohistochemistry was performed using antibodies against KRT19 (red) and KRT20 (green); DAPI was used to counterstain nuclei (C, F, I, L; blue). Each row depicts images of a single representative section. White arrows indicate examples of cells staining positive for both KRT19 and KRT20; yellow arrows indicate KRT20-positive cells that do not appear to express KRT19. Scale bar in A is for all sections (50 μm). (PDF) [file pone.0213325.s004.pdf]

Supporting Information: S4 Figure

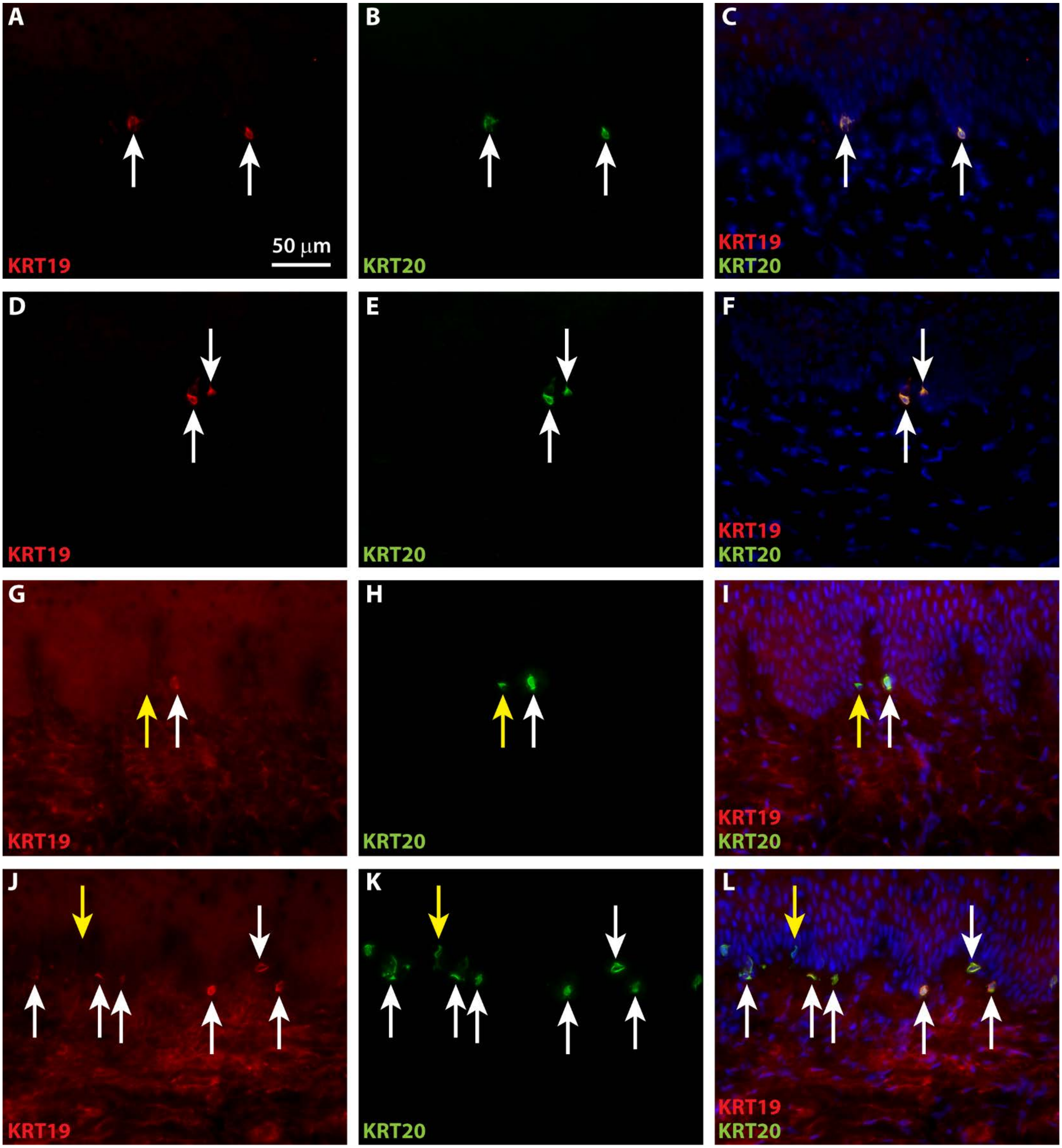

S4. Co-localization of KRT19 and KRT20 in Merkel cells in sections of ESS *in vivo*. Shown are sections of ESS excised from mice at 4 weeks (**A**-**C**), 6 weeks (**D**-**F**), 8 weeks (**G**-**I**), and 12 weeks (**J**-**L**) after grafting to mice. Immunohistochemistry was performed using antibodies against KRT19 (red) and KRT20 (green); DAPI was used to counterstain nuclei (**C**, **F**, **I**, **L**; blue). Each row depicts images of a single representative section. White arrows indicate examples of cells staining positive for both KRT19 and KRT20; yellow arrows indicate KRT20-positive cells that do not appear to express KRT19. Scale bar in **A** is for all sections (50 µm).
