# Supplementary material for: Identification of Merkel cells associated with neurons in engineered skin substitutes after grafting to full thickness wounds
Source: PLoS One. 2019 Mar 5;14(3):e0213325. doi: 10.1371/journal.pone.0213325 (PMC6400390; doi:10.1371/journal.pone.0213325)
Supplement: S5 Fig — Shown are epidermal sheets of ESS excised from mice at 2 weeks (A-C), 8 weeks (D-F), and 14 weeks (G-I), and epidermal sheets of normal human skin (J-L), stained using antibodies against KRT19 (red) and KRT20 (green); DAPI was used to counterstain nuclei (C, F, I, L; blue). Each row depicts three images of the same representative microscopic field. White arrows indicate examples of cells staining positive for both KRT19 and KRT20; yellow arrows indicate KRT20-positive cells that do not appear to express KRT19. Scale bar in A is for all sections (50 μm). (PDF) [file pone.0213325.s005.pdf]

Supporting Information: S5 Figure

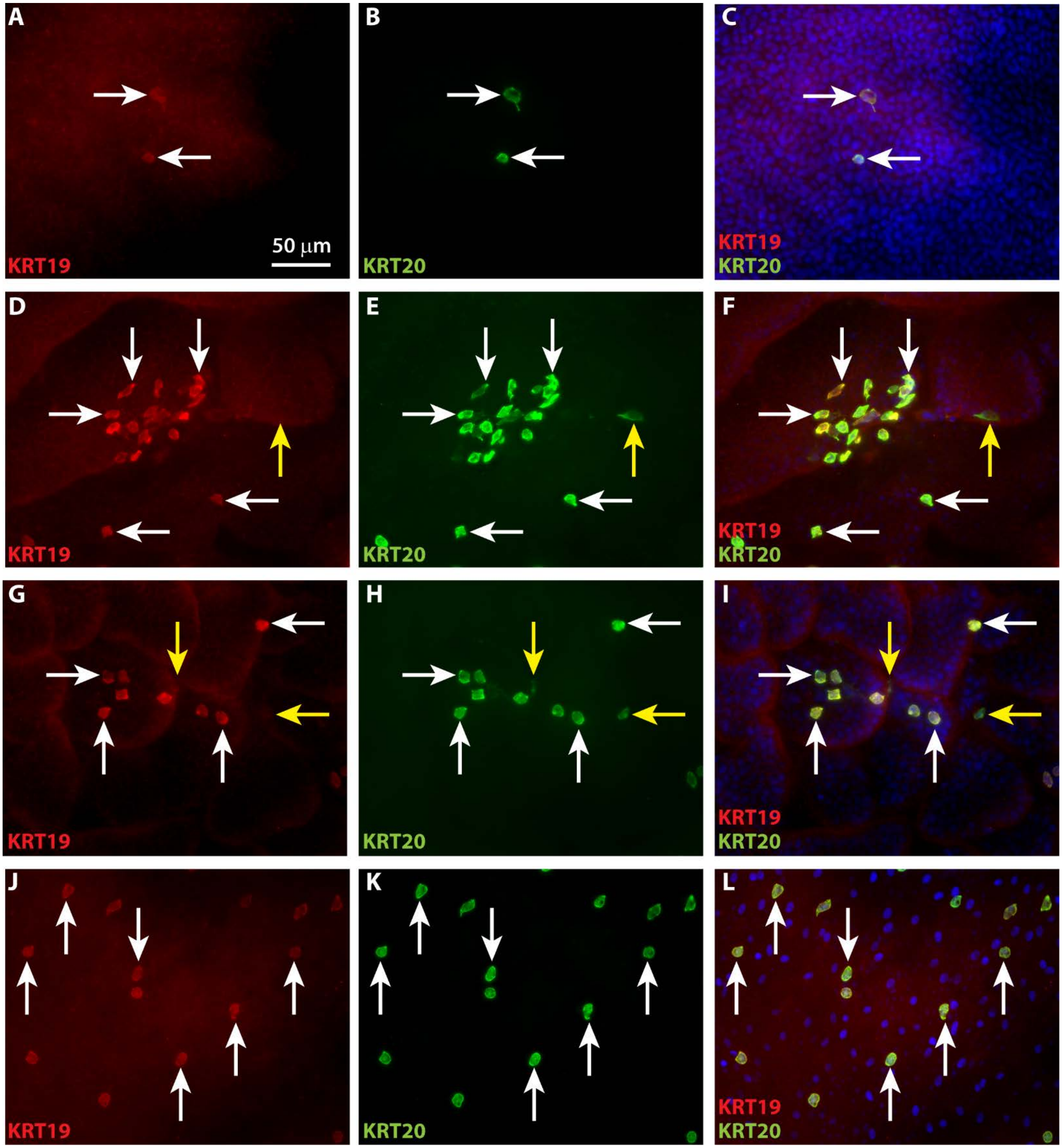

S5. Co-localization of KRT19 and KRT20 in Merkel cells in ESS epidermal sheets. Shown are epidermal sheets of ESS excised from mice at 2 weeks (**A**-**C**), 8 weeks (**D**-**F**), and 14 weeks (**G**-**I**), and epidermal sheets of normal human skin (**J**-**L**), stained using antibodies against KRT19 (red) and KRT20 (green); DAPI was used to counterstain nuclei (**C**, **F**, **I**, **L**; blue). Each row depicts three images of the same representative microscopic field. White arrows indicate examples of cells staining positive for both KRT19 and KRT20; yellow arrows indicate KRT20-positive cells that do not appear to express KRT19. Scale bar in **A** is for all sections (50 µm).
