# Supplementary material for: Identification of Merkel cells associated with neurons in engineered skin substitutes after grafting to full thickness wounds
Source: PLoS One. 2019 Mar 5;14(3):e0213325. doi: 10.1371/journal.pone.0213325 (PMC6400390; doi:10.1371/journal.pone.0213325)
Supplement: S6 Fig — Shown are en face images of ESS collected at 14 weeks after grafting. Arrows indicate examples of cells expressing both KRT20 (green) and CGA (red); DAPI (blue) was used to counterstain nuclei. (PDF) [file pone.0213325.s006.pdf]

Supporting Information: S6 Figure

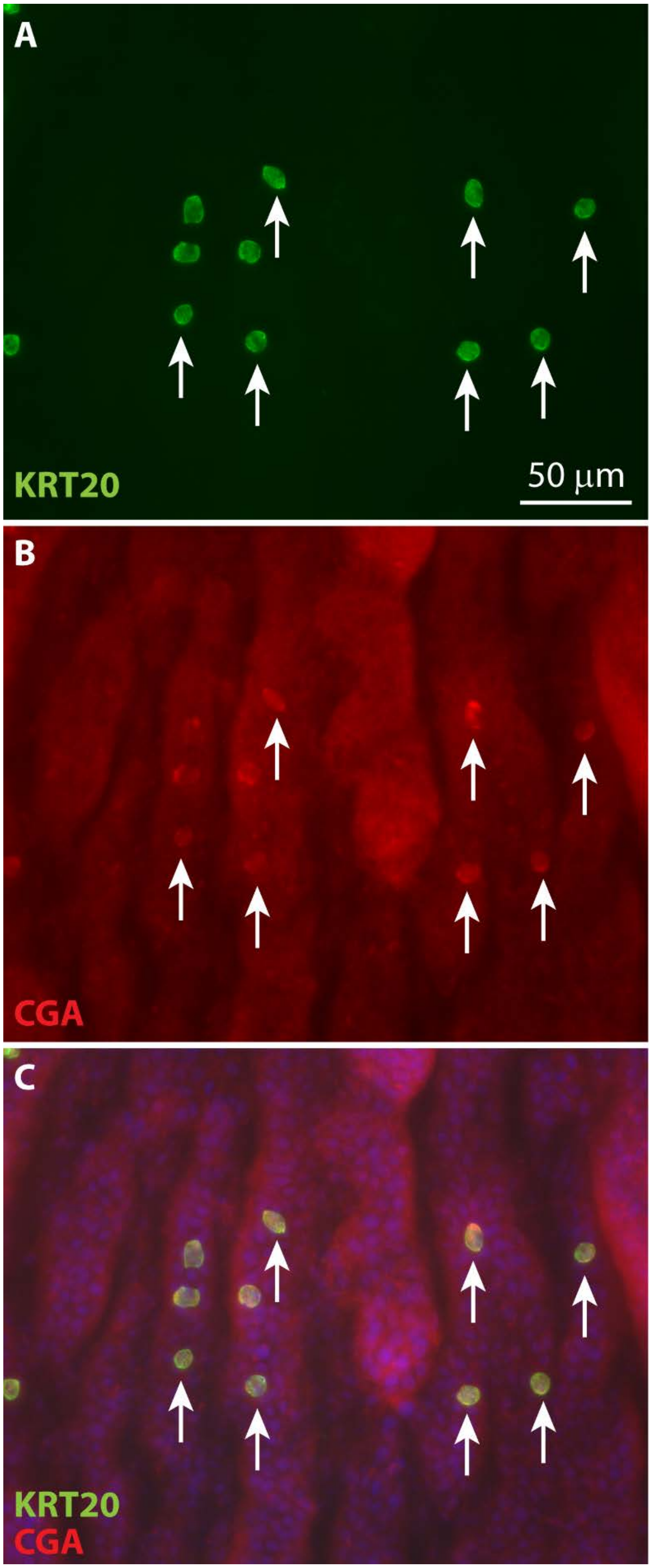

S6. Expression of neuroendocrine marker chromogranin A (CGA) in engineered skin *in vivo*. Shown are *en face* images of ESS collected at 14 weeks after grafting. Arrows indicate examples of cells expressing both KRT20 (green) and CGA (red); DAPI (blue) was used to counterstain nuclei.
